# Effectiveness and Safety of Virtual Reality Interventions for Symptom Management in Adults Undergoing Hemodialysis: Systematic Review and Meta-Analysis of Randomized Controlled Trials

**DOI:** 10.2196/102331

**Published:** 2026-09-28

**Authors:** Jiayu Deng, Wanyi Liu, Xiu Wang, Jing Hu, Hao Zhang, Jing Zeng

**Affiliations:** 1 School of Nursing, Chengdu Medical College, No. 783 Xindu Avenue, Xindu District, Chengdu, Sichuan, 610599, China, 86 13688099525

**Keywords:** virtual reality, hemodialysis, symptom management, quality of life, meta-analysis

## Abstract

**Background:**

Adults receiving long-term hemodialysis often experience a multidimensional symptom burden that affects their functional status and daily activities. Effective symptom management may help reduce this burden, preserve functional status, and improve the overall treatment experience. However, evidence remains limited for nonpharmacological interventions that are safe, acceptable, and easy to integrate into routine hemodialysis care. Virtual reality (VR) may be a promising approach for symptom relief and supportive management, but its overall effectiveness and safety in adults receiving hemodialysis remain unclear.

**Objective:**

This review aims to determine whether VR-based interventions improve symptom-related outcomes and are safe for adults receiving hemodialysis.

**Methods:**

A comprehensive search was conducted across 12 electronic databases and trial registries from inception to January 2026. Eligible studies were randomized controlled trials evaluating VR-based interventions for symptom-related outcomes in adult patients receiving hemodialysis. The methodological quality of the included trials was appraised using the Cochrane Risk of Bias 2 tool for randomized trials (RoB 2). Quantitative synthesis was conducted in RevMan 5.4 (Cochrane) when appropriate. For continuous variables, treatment effects were summarized as mean differences (MDs) or standardized mean differences (SMDs) according to outcome measurement consistency. Statistical heterogeneity was examined using the chi-square test and *I*^²^ value, and the choice between fixed-effect and random-effects models was based on the degree of heterogeneity. Outcomes unsuitable for meta-analysis were summarized descriptively.

**Results:**

Of the 11,192 records identified, 19 randomized controlled trials from 10 countries were included, involving 1228 participants. Thirteen studies used immersive VR and 6 used nonimmersive VR. Compared with usual care or no VR intervention, moderate-certainty evidence showed that VR reduced pain during arteriovenous fistula cannulation (MD −2.44, 95% CI −2.91 to −1.97; *P*<.001; *I*²=66%), improved gait speed (SMD 0.46, 95% CI 0.11-0.81; *P*=.01; *I*²=0%), increased 6-minute walk distance (MD 74.77, 95% CI 40.55-108.99; *P*<.001; *I*²=43%), and reduced depressive symptoms (MD −3.48, 95% CI −6.77 to −0.18; *P*=.04; *I*²=33%). Evidence for trait anxiety was of very low certainty (MD −7.72, 95% CI −13.72 to −1.71; *P*=.01; *I*²=61%). No serious VR-related adverse events were reported.

**Conclusions:**

Preliminary evidence suggests that VR-based interventions may reduce arteriovenous fistula cannulation pain and depressive symptoms and may improve selected physical function outcomes, including gait speed and 6-minute walk distance, in adults receiving hemodialysis. No serious VR-related adverse events were reported, although safety evidence remains limited. Further high-quality studies are warranted to confirm and refine these findings.

## Introduction

Chronic kidney disease (CKD) has emerged as a substantial contributor to the global disease burden, with approximately 788 million people affected worldwide [1]. According to a 2025 World Health Assembly (WHA) resolution, kidney disease is among the fastest-rising causes of mortality globally and may become the fifth leading cause of death by 2050 [2]. As CKD progresses, some patients develop end-stage kidney disease and require kidney replacement therapy. Hemodialysis is the most used form of kidney replacement therapy worldwide, accounting for approximately 69% of all kidney replacement therapy, and plays an important role in the long-term management of end-stage kidney disease [3]. However, patients receiving long-term hemodialysis often experience a substantial symptom burden. Due to kidney function decline and dialysis-related factors, these patients commonly report multiple symptoms, such as pruritus, fatigue, and sleep disturbance, which can affect physical function, psychological well-being, and quality of life [4,5]. A high symptom burden may impair physical function and life satisfaction and may increase the risk of adverse outcomes. In real-world hemodialysis practice, symptom management is often limited by complex comorbidities, drug interactions, and medication burden [4]. Patients receiving long-term hemodialysis require continuous, intensive treatment. Relying solely on pharmacological approaches for symptom relief may further increase treatment burden and affect adherence [6,7]. Therefore, safe and acceptable nonpharmacological interventions that can be easily integrated into the hemodialysis workflow have become an important direction for improving symptom management in this population.

Virtual reality (VR) is generally defined as a computer-generated digital environment that allows users to interact with simulated real or imagined scenarios in real time [8]. VR systems are commonly classified according to their level of immersion as nonimmersive, semi-immersive, or immersive, although health care–focused and clinical VR studies often operationalize this distinction primarily as immersive versus nonimmersive VR according to device type, interactivity, and mode of virtual environment presentation [9,10]. Immersive VR usually relies on head-mounted displays (HMDs), VR headsets, or VR goggles to provide users with a highly immersive simulated experience and a stronger sense of presence within the virtual environment by largely blocking out real-world sensory input [11]. In contrast, nonimmersive VR typically presents virtual content through external displays, such as computer screens, televisions, gaming consoles, somatosensory systems, or wearable sensor–based feedback interfaces, allowing users to interact with virtual content while remaining visually connected to the real-world environment [9,10]. VR has been widely explored across health care applications, including health professional training [12], surgical simulation and preoperative planning [13], psychotherapy [14], pain management [15], rehabilitation and exercise training [16], patient education [17], supportive care [18], and relaxation- or distraction-based interventions [19]. By enhancing presence, redirecting attention, and modulating emotional responses, VR may reduce symptom perception. Through interactive feedback and gamified tasks, it may also improve patient engagement, offering a feasible nonpharmacological approach to symptom management [20,21]. Previous studies have explored the use of VR in symptom management among adults undergoing hemodialysis, with VR-based interventions mainly concentrated in 2 clinical contexts. The first involves arteriovenous fistula cannulation in which VR is typically used as a distraction strategy to reduce cannulation-related pain, with potential benefits for anxiety, patient satisfaction, and selected hemodynamic responses [22-26]. The second involves intradialytic exercise training, in which VR is integrated into exercise programs to enhance engagement through virtual environments and interactive feedback, thereby improving physical function, walking capacity, health-related quality of life, and some psychological symptoms [27,28].

However, several limitations remain in the existing literature. First, previous studies have largely focused on VR combined with exercise training or metaverse-related technologies, with outcomes mainly centered on walking capacity, physical function, psychological status, and quality of life. Evidence has not yet been systematically synthesized with symptom management in adults undergoing hemodialysis as the primary focus [27,29,30]. Second, some studies have included both hemodialysis and peritoneal dialysis patients or have analyzed VR together with nonpure VR interventions such as sensor-based exercise gaming, thereby reducing the specificity of their conclusions for VR interventions in the hemodialysis setting [31]. Third, existing research has often focused on a single application scenario or a limited range of outcomes, with insufficient comprehensive evaluation of multidimensional outcomes such as pain, emotional symptoms, physical function, health-related quality of life, vital signs, nutritional indicators, and adverse events [22]. Finally, substantial variation remains in the implementation of VR interventions, including device type, level of immersion, and intervention dose, as well as in comparator conditions and outcome assessment, including measurement instruments and assessment time points. Together with the limited sample sizes of individual trials, these factors constrain accurate appraisal of the practical value of VR in dialysis care. This systematic review and meta-analysis synthesized randomized controlled trial (RCT) evidence on the effects of VR interventions on symptom burden, physical function, health-related quality of life, and safety in adults receiving hemodialysis.

## Methods

### Eligibility Criteria

#### Inclusion Criteria

Studies were included if they met the following criteria: (1) participants: adult patients who had received hemodialysis for at least 3 months; (2) interventions: any type of VR-based intervention delivered in the context of hemodialysis-related treatment or management; (3) comparators: the control group received usual care, no VR intervention, or other non-VR interventions; (4) primary outcomes: symptom management–related outcomes, such as fatigue, pain, sleep disturbance, anxiety, and depression; secondary outcomes included physical function outcomes, such as mobility, walking ability, and balance, and vital signs, such as blood pressure, heart rate (HR), respiratory rate (RR), and oxygen saturation; and (5) study design: RCTs.

#### Exclusion Criteria

Studies were excluded if they met any of the following criteria: (1) nonoriginal randomized controlled studies, including conference abstracts, conference presentations, case reports, and reviews; (2) interventions or group designs that did not meet the eligibility criteria; (3) outcomes outside the scope of this review, or no extractable outcome data; (4) duplicate publications or reports from the same cohort that provided no additional eligible outcome data; (5) articles not published in Chinese or English; and (6) full texts unavailable, data not extractable, or obvious data errors that could not be corrected.

### Search Strategy

This systematic review and meta-analysis followed the PRISMA (Preferred Reporting Items for Systematic Reviews and Meta-Analyses) 2020 reporting guidelines [32], and the completed checklist is available in Checklist 1. Literature retrieval was performed across major English- and Chinese-language sources. The English-language sources comprised PubMed, Embase, Web of Science, Cochrane Library, Ovid MEDLINE, Scopus, CINAHL, PsycINFO, ProQuest Dissertations & Theses Global, and ClinicalTrials.gov, while the Chinese-language sources comprised China National Knowledge Infrastructure and Wanfang Data. All databases were searched from inception to January 2026. Controlled vocabulary terms and free-text terms were combined in all searches. The search strategy was adapted for each database. In addition, citation tracking was performed to identify further eligible studies. The detailed search strategies are provided in Multimedia Appendix 1.

### Study Screening, Data Collection, and Risk-of-Bias Assessment

All retrieved citations were managed in EndNote 21 (Clarivate), where duplicate entries were identified and deleted. Two reviewers (WL and JD) performed the screening independently. Records that were clearly unrelated to the review question were excluded after title and abstract screening, and potentially relevant or uncertain records were subsequently assessed in full text. Data from eligible trials were independently collected by the same 2 reviewers. The methodological risk of bias was evaluated with the Cochrane risk of bias 2 tool for randomized trials (RoB 2) [33], which examines 5 domains: bias arising from the randomization process, bias due to deviations from intended interventions, bias related to missing outcome data, bias in outcome measurement, and bias in selection of the reported result. Domain-level judgments and the overall judgment were rated as low risk, some concerns, or high risk.

The certainty of evidence for each outcome was rated in GRADEpro (Evidence Prime) according to the GRADE (Grading of Recommendations Assessment, Development and Evaluation) approach, with evidence categorized as high, moderate, low, or very low. The rating process considered study design, risk of bias, inconsistency, indirectness, imprecision, and publication bias [34]. Any discrepancies were settled through discussion; when agreement was not achieved, a senior researcher (HZ) made the final decision.

### Data Extraction

For classification in this review, interventions delivered through HMDs, VR headsets, or VR goggles were classified as immersive VR, whereas interventions delivered through external screens, gaming consoles, somatosensory systems, or wearable sensor–based feedback interfaces were classified as nonimmersive VR.

For multiple reports from the same research team or research program, potential cohort overlap was assessed by comparing author group, study setting, sample size, participant characteristics, intervention duration, comparator condition, outcome measures, and author descriptions. Reports that appeared to arise from the same or potentially overlapping cohort were treated as linked reports. To avoid double counting, overlapping participant data were not included more than once in the same outcome-specific meta-analysis. When linked reports provided different eligible outcomes, only the relevant nonoverlapping outcome data were extracted for each analysis, and sensitivity analyses were conducted excluding these studies when feasible.

For standard 2-arm parallel-group RCTs, data were extracted from the VR intervention group and the corresponding comparator group for each eligible outcome. Comparator groups were classified according to their clinical content as usual care, no additional intervention, or active non-VR intervention. Active non-VR comparators, such as aromatherapy, conventional exercise training, nurse-supervised exercise, or neuromuscular electrical stimulation, were not considered clinically equivalent to usual care. For multiarm, factorial, or crossover trials, data were extracted according to the comparison most relevant to the VR component. Only one eligible comparison was included in each meta-analysis, shared groups were not reused, and first-period data were used for crossover trials when paired treatment effects were not fully extractable.

### Statistical Analysis

Quantitative synthesis was conducted in RevMan 5.4 (Cochrane). For continuous variables, the summary effect was chosen according to whether outcome measurements were comparable across studies. When the same instrument or measurement unit was used, results were pooled as mean differences (MDs). When outcomes were assessed using different instruments or scales, standardized mean differences (SMDs) were calculated. All pooled estimates were presented with 95% CIs. Specifically, statistical significance was defined using a 2-sided significance level of .05, and the heterogeneity test was considered nonsignificant at the .10 level for model selection. Between-study heterogeneity was assessed using the chi-square test and the *I*² statistic. When *P*≥.10 and *I*²<50%, a fixed-effect model was used. Otherwise, a random-effects model was applied. Considering the anticipated clinical and methodological heterogeneity across studies, model-based sensitivity analyses were additionally performed by recalculating each pooled outcome using the alternative model.

If sufficient studies were available, subgroup analyses were planned according to clinically relevant factors, including VR immersion level, application scenario, comparator type, and intervention duration. Sensitivity analyses were performed to examine the robustness of pooled results. For outcomes with at least 3 studies, leave-one-out sensitivity analyses were performed by sequentially excluding one study at a time and recalculating the pooled effect estimate. Model-based sensitivity analyses were summarized in the supplementary materials. Studies that could not be quantitatively pooled were summarized narratively. In accordance with the Cochrane Handbook, publication bias was assessed using funnel plots or statistical tests only when at least 10 studies were available for a given outcome [35].

## Results

### Study Selection

The database search yielded 11,192 records, of which 519 were retrieved from Chinese-language databases and 10,673 from English-language databases. After removal of duplicate records, 9262 citations were retained for screening. Title and abstract review led to 126 reports being selected for full-text evaluation. Ultimately, 19 articles met the inclusion criteria, including 2 Chinese-language articles and 17 English-language articles. Details of the selection process are presented in Figure 1. Reports with unavailable, incomplete, or unsuitable data identified during study selection are listed in Table S1 in Multimedia Appendix 2, with study-level reasons for noninclusion in the meta-analysis.

**Figure 1.**
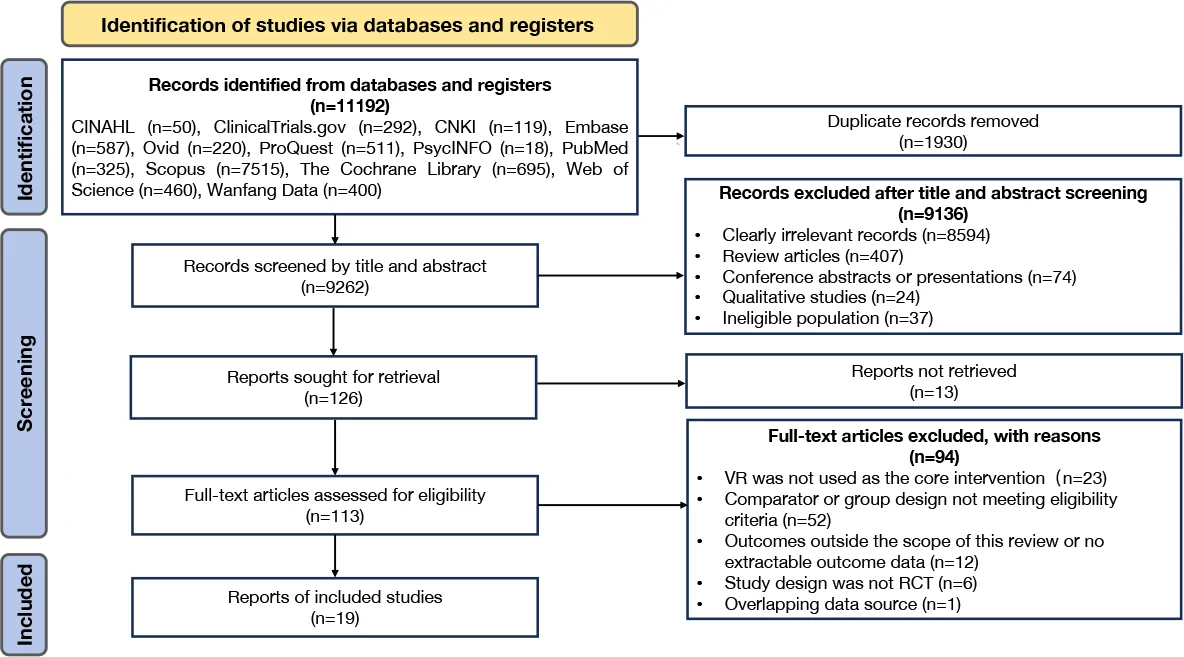
PRISMA (Preferred Reporting Items for Systematic Reviews and Meta-Analyses) flow diagram of study selection. RCT: randomized controlled trial; VR: virtual reality.

### Basic Characteristics of Included Studies

Data extraction covered the first author, year of publication, country or region, study design, sample size, intervention characteristics, intervention length, and reported outcomes. Table 1 presents the main features of the included studies and the corresponding VR intervention protocols. A total of 19 reports were included. After accounting for linked reports from the same research programs, these reports represented 16 independent or nonoverlapping parent cohorts, with 973 unique participants. Reports with potentially overlapping cohorts were retained only when they contributed distinct outcome domains, and duplicated participant data or overlapping outcomes were not counted more than once. The number of participants in each study varied from 10 to 93. These studies were conducted across 10 countries: Poland contributed 4 studies [36-39]; Turkey [23,26,40] and Spain [41-43] contributed 3 studies each; China [44,45] and Iran [24,46] contributed 2 studies each; and the United States [47], Germany [48], Egypt [25], Brazil [49], and Qatar [50] contributed 1 study each.

**Table 1.** Characteristics of the included studies^a^.

Author (year); country	Study design	Participants	Technology	Intervention		Control	Main outcomes
				Type	Details		
Güler et al [26] (2026); Turkey	RCT^b^	n=80 VR^c^ group: 40 (female 14, male 26; age (mean 43.25, SD 6.48 y) Control group: 40 (female 13, male 27; age (mean 45.15, SD 7.62 y)	VR-Box 3D glasses + Android smartphone, supports 360° VR content; headphones for audio immersion	Immersive VR system	Duration: single session (10 min) Frequency: once (during 1 dialysis treatment) Follow-up: not reported Adverse event: 4 patients in intervention group removed VR glasses due to discomfort	Usual care	Physiological indicators: pulse rate, SBP^d^, DBP^e^, SPO_2_^f^ Pain: VAS^g^ STAI^h^
Mohamed Elzeky et al [25] (2024); Egypt	RCT	n=93 VR group: 47 (female 23, male 24); age (mean 48.9, SD 11.1 y) Control group: 46 (female 21, male 25); age (mean 47.0, SD 12.3 y)	VR BOX 2.0 glasses with a smartphone and headphones; preloaded 360° Relax River VR Tour video	Immersive VR system	Duration: single session (6 min total) Frequency: once (during 1 HD^i^ session) Implementation: 6 min before AVF^j^ puncture, continued during the puncture procedure Follow-up: not reported Adverse event: not reported	Usual care	Hemodynamic: HR^k^, SBP, DBP, RR^l^, SPO_2_ Pain: VAS Anxiety: STAI-S^m^ Satisfaction: VAS
Heirati et al [46] (2025); Iran	RCT; 2×2 factorial design	n=73 AR^n^ group: 20 (female 11, male 9) VR group: 19 (female 8, male 11) AR + VR group: 17 (female 6, male 11) Control group: 17 (female 9, male 8) baseline sample mean age 55.19 y (n=80)	AR group: rose essential oil VR group: immersive 3D VR glasses AR + VR: VR glasses + rose aroma inhalation	Immersive VR+ rose aroma inhalation	Duration: 4 wk Frequency: once a week, 15 min/session Implementation: 20 min post-HD; AR + VR group simultaneously uses VR and inhales rose aroma; AR group only inhales rose aroma; VR group only uses VR Follow-up: not reported Adverse event: 1 VR group patient withdrawn due to eye pain; 3 AR + VR group patients withdrawn (1 dissatisfaction, 2 illness); 3 control group patients withdrawn (dissatisfaction)	Usual care	Anxiety: STAI-S and STAI-T^o^ Happiness: OHQ^p^ Sleep quality: PSQI^q^
Coşar and Bingöl [40] (2026); Turkey	RCT; pretest-posttest design	n=50 VR group: 25 (female 14, male 11); age (mean 3.72, SD 8.73 y) Control group: 25 (female 10, male 15); age (mean 59.96, SD 11.14 y)	Dialysis-based VR video distraction intervention	Immersive VR	Duration: 8 wks Frequency: twice a week, 30 min/session (5 min after HD start) Follow-up: not reported Adverse event: not reported	Usual care	Dialysis symptoms: DSI^r^ Comfort: HDCS-II^s^ Hemodynamic: HR, SBP, DBP, RR, SPO_2_
Maynard et al [49] (2019); Brazil	RCT	n=40 VR group: 20 (female 10, male 10); age (mean 43.9, SD 11.7 y) Control group: 20 (female 8, male 12); age (mean 49, SD 15.2 y)	Nonimmersive VR system (Nintendo Wii console + Wii Sports/Wii Fit Plus software, including various sports games such as Penguin Slide and Soccer Heading); assistive equipment (stationary bike dynamometer, Thera-Band resistance bands, 1‐2 kg ankle weights)	Exercise training during HD combined with nonimmersive VR intervention	Duration: 12 wk Frequency: 3 times/wk, conducted during the first 2 h of HD; session duration gradually increased from 30‐40 min to 55‐60 min Follow-up: assessment conducted at the end of the 12-wk intervention Adverse events: not reported	Usual care	Functional capacity: TUG^t^, 10 m walking speed, DASI^u^ Health-related quality of life: KDQOL-SF^v^ Depressive symptoms: CES-D^w^ Scale Hemodynamic: HR, RR, SBP, DBP, SpO₂
He Zhou et al [50] (2020); Qatar	RCT	n=73 VR group: 37 (female 18, male 19); age (mean 62.7, SD 6.8 y) Control group: 36 (female 22, male 14); age (mean 66.5, SD 10.0 y)	Wearable sensor-based intradialytic exergame system (LEGSys sensors + interactive laptop interface)	Nonimmersive VR system	Duration: 4 wk Frequency: 3 sessions/wk (for total of 12 exercise sessions, 30 min/session) Follow-up: not reported Adverse event: none reported	Nurse-supervised intradialytic exercise	Depressive symptoms: CES-D User experience: TAM^x^
Martínez-Olmos et al [41] (2022); Spain	Crossover RCT	n=33 Sequence A: VR → Control, n=15; age (mean 66.5, SD 14.8 y) Sequence B: Control → VR, n=18; age (mean 68.0, SD 13.5 y)	Nonimmersive VR system (Microsoft Kinect motion tracking camera, standard computer, TV screen); adapted “Treasure Hunt” VR video game	Nonimmersive VR system	Duration: 12 wk (intervention period), total study duration 24 wk Frequency: 3 times/wk, up to 40 min/session Follow-up: not reported Adverse event: none reported	Usual care	Physical function: 4 m gait speed test, SPPB^y^, TUG, OLST^z^, STS-5^aa^/STS-10^ab^/STS-60^ac^, 6MWT^ad^ Exercise adherence: percentage of completed sessions
Schinner et al [48] (2023); Germany	Multicenter prospective RCT	n=32 NMES^ae^ group: 12 (female 2, male 10); age (mean 66.75, SD 9.81 y) NMES + VR group: 9 (female 1, male 8); age (mean 66.22, SD 9.11 y) Control group: 11 (female 3, male 8); age (mean 72.00, SD 11.39 y)	4-channel NMES device (STIM-PRO X9+), immersive VR headset (Oculus Go) with relaxation, journey, or interactive game apps	Immersive VR	Duration: 12 wk Frequency: 2‐3 times/wk, total 60 min/wk Follow-up: assessed at baseline (t0) and 12 wk (t1) Adverse event: 1 patient in NMES group reported cramps; no serious adverse events related to interventions	Usual care	Muscle strength: isometric knee extensor strength (quadriceps femoris muscle) Functional capacity: STS-60 Serum biochemistry: SAlb^af^, creatine kinase Body composition: weight, BMI, muscle circumference, fat, water, and muscle proportion
Turoń-Skrzypińska et al [36] (2023); Poland^ag^	RCT	n=85 VR group: 39 (female 10, male 29); age (mean 57.56, SD 17.61 y) Control group: 46 (female 17, male 29); age (mean 62.63, SD 15.47 y)	VR exercise training during HD using NefroVR system (cycling with audiovisual stimulation, 5 mini-games, ~20-min duration, intensity guided by Borg scale 8‐14)	Immersive VR	Duration: 3 months Frequency: 3 times/wk, 20 min/session (conducted during first 1‐2 h of HD or until UF 2.5 is achieved) Follow-up: assessed at baseline (E0) and 3 months (E3) Adverse event: not reported	Usual care	Depression symptoms: BDI^ah^ Anxiety symptoms: GAD-7^ai^ questionnaire
Turoń-Skrzypińska et al [37] (2024); Poland^ag^	Prospective cohort study (RCT design with random assignment)	n=85 VR group: 39 (female 10, male 29); age (mean 57.56, SD 17.61 y) Control group: 46 (female 17, male 29); age (mean 62.63, SD 15.47 y)	VR exercise training during HD using NefroVR system (cycling with audiovisual stimulation, 5 mini-games~20 min duration, intensity guided by Borg scale 8‐14)	Immersive VR	Duration: 3 months Frequency: 3 times/wk, 20 min/session (first 2 h of HD or until UF 2.5) Follow-up: baseline (E0) and 3 months (E3) Adverse events: not reported	Usual care	Level of physical activity: IPAQ^aj^ Physical capacity: 6MWT Blood sampling: IL-6^ak^, human SOST^al^
Turoń-Skrzypińska et al [38] (2025); Poland^ag^	RCT	n=85 VR group: 39 (female 10, male 29); age (mean 57.56, SD 17.61 y) Control group: 46 (female 17, male 29); age (mean 62.63, SD 15.47 y)	VR exercise training during HD using NefroVR system (cycling with audiovisual stimulation, 5 mini-games~20 min duration, intensity guided by Borg scale 8‐14)	Immersive VR	Duration: 3 months Frequency: 3 times/wk, 20 min/session (first 2 h of HD or until UF 2.5) Follow-up: baseline (E0) and 3 months (E3) Adverse events: not reported	Usual care	Inflammatory marker: TNF-α^am^ concentration Lipid metabolism marker: PCSK9^an^ concentration Safety: Borg scale
Eva Segura‐Ortí et al [42] (2019); Spain^ao^	RCT	n=40 VR group: 20 Control group: 20	Intradialytic VR exercise (Treasure Hunt game with progressive duration from 3 to 6 min per bout, total up to 36 min; warm-up and cool-down 5 min each; intensity “somewhat hard” on Borg scale; difficulty automatically adjusted by system based on performance)	Nonimmersive VR system	Duration: 12 wk Frequency: 3 times/wk during HD progression from 3×3 min up to 6×6 min Follow-up: baseline and 12 wk Adverse events: not reported	Usual care	Physical function: STS-10, STS-60, gait speed, handgrip, 6MWT Physical activity: HAP AAS^ap^ HRQoL:^aq^ PF^ar^, VT^as^ Hemodynamic safety: BP^at^, HR
Eva Segura‐Ortí et al [43] (2019); Spain ^ao^	Feasibility randomized trial	n=18 VR group: 9 (female 3, male 6); age (mean 68.3, SD 15.6 y) Control group: 9 (female 4, male 5); age (mean 61.8, SD 13.0 y)	Nonimmersive VR system (computer, TV, and Microsoft Kinect motion tracker) with “A la caza del tesoro” (Treasure Hunt) game where patients move legs to catch coins and avoid bombs	Nonimmersive VR system	Duration: 4 wk (after 16 wk combined exercise) Frequency: 3 times/wk, up to 30 min/session Adverse events: not reported	Conventional exercise training	Physical function: STS-10, STS-60, gait speed, OLHR^au^, 6MWT. Adherence rate (%)
Namazinia et al [24] (2025); Iran	RCT	n=60 VR group: 30 (female 14, male 16); age (mean 50.0, SD 6.8 y) Control group: 30 (female 13, male 17); age (mean 51.1, SD 6.4 y)	Shinecon 4th Gen VR Headset with smartphone displaying 360° nature videos	Immersive VR	Duration: 5 min prior to AVF puncture + the entire puncture procedure Frequency: one session per patient Follow-up: not reported Adverse events: not reported	Usual care	Pain: VAS
Burrows et al [47] (2023); United States	Feasibility randomized controlled trial	n=10 VR + PARx^av^ group: 6 (female 4, male 2); age (mean 59.67, SD 14.12 y) PARx group: 4 (female 3, male 1); age (mean 59.50, SD 15.07 y)	Oculus Quest 2 head-mounted display with JovialityTM (custom VR mindfulness program) and Guided Meditation VR app (29 virtual environments: beach, mountains, forest trail, and so on)	Immersive VR mindfulness + personalized intradialytic exercise	Duration: total 10 wk (2-wk VR mindfulness+8-wk personalized exercise) Frequency: 3 sessions/wk during HD Follow-up: baseline, post-prehabilitation (2 wk), postintervention (10 wk) Adverse event: not reported	PARx alone	Feasibility: recruitment, retention, adherence, and acceptability Depressive symptoms: PROMIS^aw^ Depression-SF8a Mindfulness: FFMQ-SF^ax^ Fatigue: SONG-HD^ay^ Physical activity energy expenditure: LoPAQ^az^
Turoń-Skrzypińska et al [39] (2023); Poland ^ag^	RCT	n=85 VR group: 39 (female 10, male 29); age (mean 57.56, SD 17.61 y) Control group: 46 (female 17, male 29); age (mean 62.63, SD 15.47 y)	VR exercise training during HD using NefroVR system (cycling with audiovisual stimulation, 5 mini-games~20 min duration, intensity guided by Borg scale 8‐14)	Immersive VR	Duration: 3 months Frequency: 3 times/wk, 20 min/session (conducted during first 1‐2 h of HD or until UF 2.5 is achieved) Follow-up: assessed at baseline (E0) and 3 months (E3) Adverse event: not reported	Usual care	Proinflammatory cytokines: plasma IL-1β^ba^, IL-6, IL-8^bb^ concentrations Safety monitoring: BP, HR, Borg scale
Feng et al [44] (2021); China	RCT	n=42 VR group: 21 (female 7, male 14); age (mean 41.00, SD 10.31 y) Control group: 21 (female 6, male 15); age (mean 37.19, SD 10.11 y)	VR-based somatosensory game (BL00M app) on smartphone	Nonimmersive VR	Duration: 6 months Frequency: 3 times/wk, 15‐30 min/session (home-based) Follow-up: baseline, 3 months, 6 months Adverse events: not reported	Usual care	Physical self-efficacy: SEE Risk of falling: Morse Fall Scale Quality of life: SF-36^bc^
Li et al [45] (2022); China	RCT	n=70 VR group: 35 (female 16, male 19); age (mean 56.9, SD 1.1 y) Control group: 35 (female 17, male 18); age (mean 56.5, SD 0.9 y)	VR headset (immersive VR); customized virtual scenes and games	Immersive VR	Duration: not explicitly stated Frequency: per HD^i^ session Follow-up: not reported Adverse events: not reported	Usual care	Anxiety: HAMA^bd^ Depression: HAMD^be^ Quality of life: SF-36 Treatment compliance
Şen H and Lafcı Bakar D [23] (2024); Turkey	RCT	n=60 VR group: 30 (female 10, male 20); age (mean 54.03, SD 15.78 y) Control group: 30 (female 9, male 21); age (mean 49.10, SD 13.48 y)	VR glasses (immersive VR); 360° video	Immersive VR	Duration: single session (2 min before AVF cannulation +3 min during puncture) Frequency: one-time per patient Follow-up: no follow-up Adverse event: not reported	Usual care	Pain: VAS Hemodynamic: HR, RR, SBP, DBP, SpO₂ Patient satisfaction

a n refers to the number of participants included in the final analysis of each report.

b RCT: randomized controlled trial.

c VR: virtual reality.

d SBP: systolic blood pressure.

e DBP: diastolic blood pressure.

f SpO₂: peripheral oxygen saturation.

g VAS: Visual Analogue Scale.

h STAI: State-Trait Anxiety Inventory.

i HD: hemodialysis.

j AVF: arteriovenous fistula.

k HR: heart rate.

l RR: respiratory rate.

m STAI-S: State-Trait Anxiety Inventory–State.

n AR: augmented reality.

o STAI-T: State-Trait Anxiety Inventory–Trait.

p OHQ: Oxford Happiness Questionnaire.

q PSQI: Pittsburgh Sleep Quality Index.

r DSI: Dialysis Symptom Index.

s HDCS-II: Hemodialysis Comfort Scale Version II.

t TUG: Timed Up and Go test.

u DASI: Duke Activity Status Index.

v KDQOL-SF: Kidney Disease Quality of Life Short Form.

w CES-D: Center for Epidemiologic Studies Depression Scale.

x TAM: technology acceptance model.

y SPPB: Short Physical Performance Battery.

z OLST: one-legged stance test.

aa STS-5: 5-times Sit-to-Stand test.

ab STS-10: 10-times Sit-to-Stand test.

ac STS-60: 1-minute Sit-to-Stand test.

ad 6MWT: 6-Minute Walk Test.

ae NMES: neuromuscular electrical stimulation.

af SAlb: serum albumin.

ag Reports were treated as linked reports from the same research program; these reports were used only for distinct outcome data, and overlapping data were not double-counted within the same outcome-specific meta-analysis.

ah BDI: Beck Depression Inventory.

ai GAD-7: Generalized Anxiety Disorder-7.

aj IPAQ: International Physical Activity Questionnaire.

ak IL-6: interleukin-6.

al SOST: sclerostin.

am TNF-α: tumor necrosis factor-α.

an PCSK9: proprotein convertase subtilisin/kexin type 9.

ao Reports were assessed for potential overlap, and no clear evidence of participant overlap was identified based on the available study information.

ap HAP AAS: Human Activity Profile Adjusted Activity Score.

aq HRQoL: health-related quality of life.

ar PF: physical function.

as VT: vitality.

at BP: blood pressure.

au OLHR: one-leg heel-rise test.

av PARx: personalized activity prescription.

aw PROMIS: Patient-Reported Outcomes Measurement Information System.

ax FFMQ-SF: Five Facet Mindfulness Questionnaire–Short Form.

ay SONG-HD: Standardized Outcomes in Nephrology–Hemodialysis.

az LoPAQ: Low Physical Activity Questionnaire.

ba IL-1β: interleukin-1β.

bb IL-8: interleukin-8.

bc SF-36: 36-item Short Form Health Survey.

bd HAMA: Hamilton Anxiety Rating Scale.

be HAMD: Hamilton Depression Rating Scale.

The included VR interventions varied by technology type, application scenario, and intervention duration. Thirteen studies used immersive VR [23-26,36-40,45-48], and 6 used nonimmersive VR [41-44,49,50]. Intervention settings varied: 13 studies delivered VR during hemodialysis [36-43,45,47-50], 4 during arteriovenous fistula cannulation [23-26], 1 at home [44], and 1 after dialysis sessions [46]. Four studies [23-26] used VR as a distraction strategy during arteriovenous fistula cannulation and mainly assessed cannulation-related pain. The remaining studies mainly integrated VR into intradialytic exercise training, home-based exercise management, or psychological support. Intervention duration generally ranged from 4 weeks to 3 months, with a few studies extending to 6 months. Most studies used a parallel-group or crossover randomized controlled design. One study [48] used a 3-arm design, and one study [46] used a 2×2 factorial design. No included study reported serious VR-related adverse events. A small number of studies reported withdrawal or discontinuation due to device-related discomfort, eye pain, lack of interest, or muscle cramps related to neuromuscular electrical stimulation.

### Quality Assessment of Included Studies

The risk of bias in the included studies was assessed using the Cochrane RoB 2 tool, and the results are shown in Figure 2. Overall, the included studies were judged mainly as having a low risk of bias or some concerns. Most studies had a relatively clear randomization process. However, some studies were judged as having some concerns because random sequence generation or allocation concealment was not sufficiently described [23,37-40,44-46]. The overall risk of bias was low for deviations from intended interventions and missing outcome data. The main concerns were related to outcome measurement and selective reporting. Some studies did not clearly report whether outcome assessors were blinded [26,36-40,42,46-48], which may increase the risk of measurement bias, especially for patient-reported outcomes. Some studies did not provide trial registration information or a prespecified analysis plan [39,44,45,47], indicating a potential risk of selective reporting bias.

**Figure 2.**
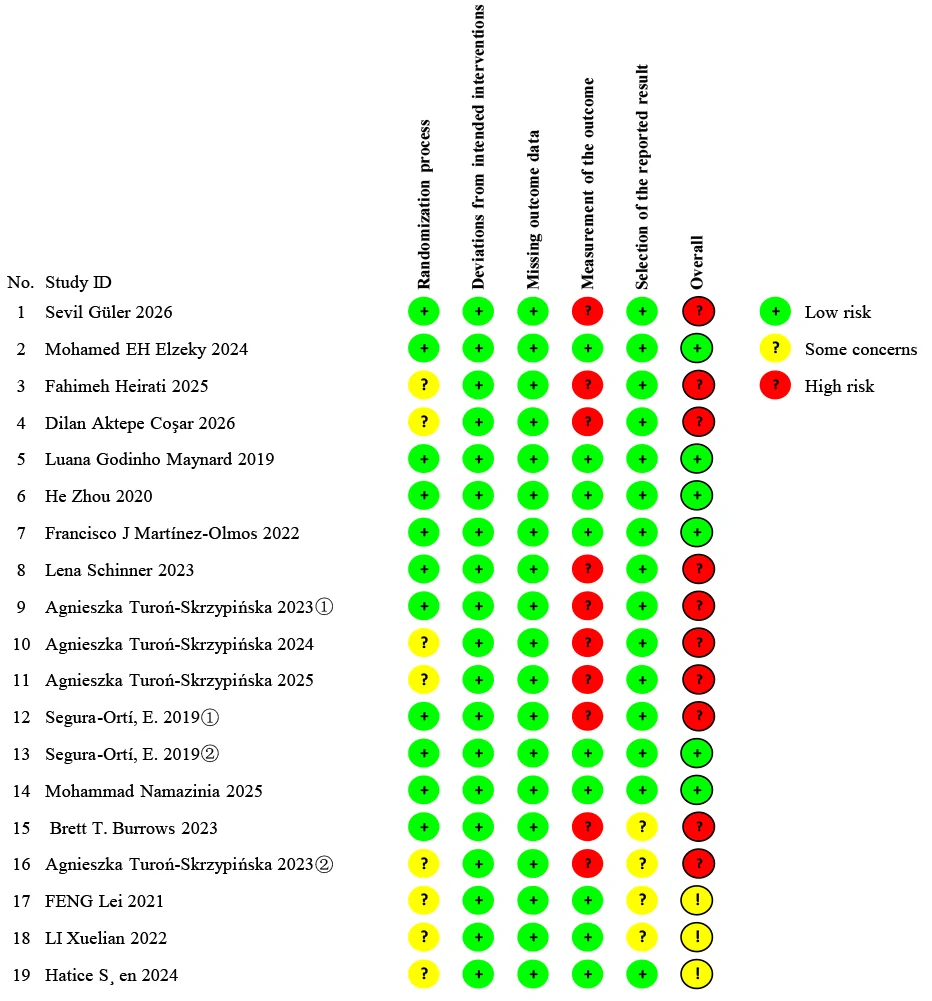
Risk-of-bias assessment of included studies [23-26,36-50].

The certainty of evidence assessed using the GRADE approach is presented in Multimedia Appendix 3. Moderate-certainty evidence was found for gait speed, arteriovenous fistula cannulation pain, depressive symptoms, Timed Up and Go test (TUG), 5-times Sit-to-Stand test (STS-5), and 6-Minute Walk Test (6MWT). Low-certainty evidence was found for HR, diastolic blood pressure (DBP), peripheral oxygen saturation (SpO2), 10-times Sit-to-Stand test (STS-10), and most health-related quality-of-life outcomes. Very low-certainty evidence was found for state anxiety, trait anxiety, 1-minute Sit-to-Stand test (STS-60), RR, and systolic blood pressure (SBP). Downgrading was mainly due to small sample sizes, heterogeneity, unstable sensitivity analyses, and risk of bias.

### Qualitative Synthesis

By summarizing the outcomes reported in the included studies, we grouped the findings into 5 themes: pain and dialysis-related discomfort, psychological health, physical function and exercise capacity, physiological and biochemical indicators, and quality of life and intervention experience. Outcomes that could not be quantitatively pooled were synthesized narratively. These outcomes mainly included dialysis-related symptoms, comfort, procedural satisfaction, sleep quality, happiness, mindfulness, fatigue, self-efficacy, fall risk, physical activity level, muscle strength, body composition, and inflammatory markers.

Narrative evidence suggested that VR interventions may reduce overall discomfort during dialysis and improve patient comfort [40] and procedural satisfaction [25]. VR combined with aromatherapy may improve sleep quality and happiness, whereas VR alone did not show a clear effect [46]. Some studies also suggested that VR may improve mindfulness and fatigue [47]. For physical function, VR-based exercise or somatosensory games may increase physical activity levels [49], enhance exercise self-efficacy, and reduce fall risk [44]. These interventions may also have positive effects on local muscle strength [48] and handgrip strength [42], although their effect on body composition remains unclear [48]. Four studies by Turoń-Skrzypińska and colleagues [36-39] found that levels of IL-6, IL-1β, IL-8, TNF-α, PCSK9, and sclerostin (SOST) decreased after VR-based exercise. These findings suggest that VR combined with exercise training may influence inflammatory and bone metabolism–related markers.

Overall, narrative evidence suggests that VR may have potential effects on symptom experience, psychological status, functional activity, and selected biochemical indicators. However, most outcomes were reported by single studies or only a small number of studies. Measurement tools, intervention formats, and data reporting methods also varied substantially. Therefore, these findings should be interpreted with caution. Outcomes summarized narratively but not quantitatively pooled are detailed in Table S2 in Multimedia Appendix 2.

### Meta-Analysis Results

To examine whether the pooled results were influenced by model choice, we performed model-based sensitivity analyses by recalculating each outcome using the alternative statistical model. The full results comparing fixed-effect and random-effects models are presented in Table S1 in Multimedia Appendix 4. In the main text, model-based sensitivity analyses are described only for outcomes whose interpretation changed after model switching. For analyses including at least 3 studies, a “leave-one-out” sensitivity analysis was further conducted to examine the impact of a single study on the pooled effect size; the complete tables and figures are provided in Multimedia Appendix 5.

### Effects of VR Interventions on Arteriovenous Fistula Cannulation Pain

Four studies [23-26] assessed the effect of VR interventions on arteriovenous fistula cannulation pain using the Visual Analogue Scale (VAS). The MD was used as the pooled effect measure. The heterogeneity test showed moderate heterogeneity across studies (*I*²=66%; *P*=.03). Therefore, a random-effects model was used. The pooled results showed that, compared with usual care, VR interventions significantly reduced arteriovenous fistula cannulation pain scores in patients undergoing hemodialysis (MD −2.44, 95% CI −2.91 to −1.97; *P*<.001; Figure 3).

**Figure 3.**
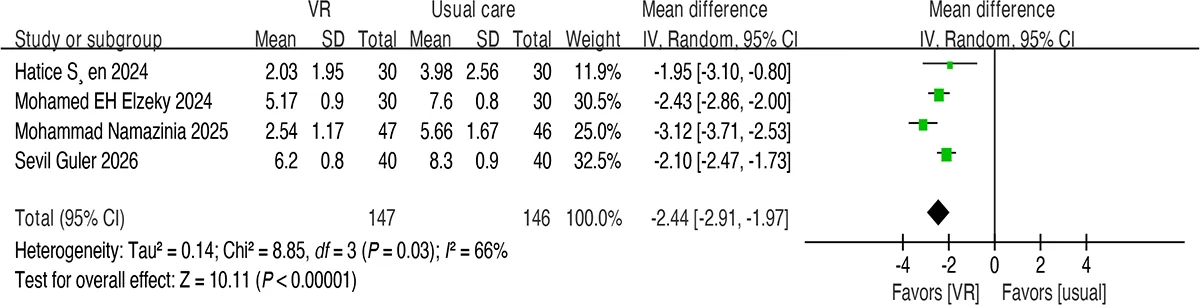
Forest plot of the effect of virtual reality intervention on arteriovenous fistula cannulation pain [23-26]. VR: virtual reality.

Sensitivity analysis showed that, after excluding the study by Namazinia et al [24], heterogeneity disappeared among the remaining 3 studies (*I*²=0%; *P*=.47) [23,25,26]. The pooled effect remained statistically significant (MD −2.22, 95% CI −2.50 to −1.95; *P*<.001). The direction and magnitude of the effect were like those of the primary analysis, suggesting that the pooled result was relatively robust. Because the number of included studies was limited, no further subgroup analysis was conducted to explore the source of heterogeneity (the sensitivity analysis forest plot is provided in Figure S1 in Multimedia Appendix 5)

### Effects of VR Interventions on Psychological Status

#### Anxiety Symptoms

Five studies assessed anxiety symptoms [25,26,36,45,46]. Because the anxiety measurement tools were not fully consistent, meta-analysis was performed only for 3 studies [25,26,46] that used the State-Trait Anxiety Inventory (STAI). The results were pooled separately for the State-Trait Anxiety Inventory–State (STAI-S) and the State-Trait Anxiety Inventory–Trait (STAI-T). The other 2 studies used the Generalized Anxiety Disorder-7 (GAD-7) [36] and the Hamilton Anxiety Rating Scale (HAMA) [45], respectively. These studies were not included in the quantitative synthesis and were summarized narratively. Both studies suggested lower anxiety scores after VR interventions compared with the control groups.

#### State Anxiety

Three studies [25,26,46] assessed state anxiety using the STAI-S. The MD was used as the pooled effect measure. The heterogeneity test showed substantial heterogeneity across studies (*I*²=98%; *P*<.001). Therefore, a random-effects model was used. The pooled results showed no statistically significant difference in STAI-S scores between the VR intervention and usual care groups (MD −9.64, 95% CI −24.43 to 5.15; *P*=.20; Figure 4). Leave-one-out sensitivity analysis showed that, after excluding the study by Güler et al [26], heterogeneity decreased markedly among the remaining studies (*I*²=4%; *P*=.31). After exclusion of this study, the pooled estimate was statistically significant (MD −4.51, 95% CI −7.60 to −1.41; *P*=.004; Figure S2 in Multimedia Appendix 5). This finding suggests that the pooled estimate and observed heterogeneity may have been influenced by this individual study and should therefore be interpreted cautiously. However, because only a small number of studies were included, the robustness of this finding remains limited. In addition, the model-based sensitivity analysis showed that the interpretation of the pooled result for state anxiety changed after switching the statistical model, indicating that this finding was dependent on model choice. Therefore, current evidence does not support a robust effect of VR interventions on state anxiety.

**Figure 4.**
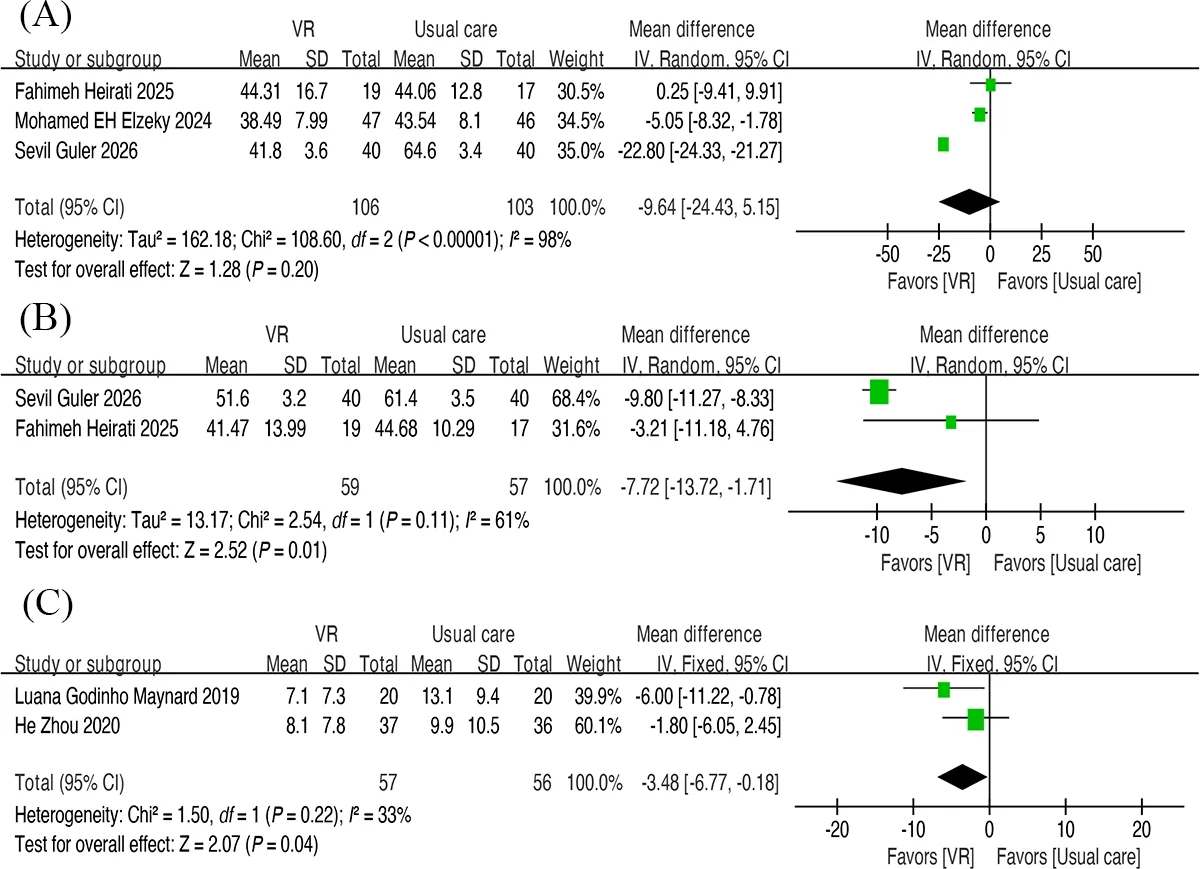
Forest plots of the effects of virtual reality (VR) interventions on anxiety and depressive symptoms. (A) Forest plot of the effect of VR interventions on state anxiety [25,26,46]. (B) Forest plot of the effect of VR interventions on trait anxiety [26,46]. (C) Forest plot of the effect of VR interventions on depressive symptoms [49,50].

#### Trait Anxiety

Two studies [26,46] assessed trait anxiety using the STAI-T. The MD was used as the pooled effect measure. The heterogeneity test showed moderate heterogeneity across studies (*I*²=61%; *P*=.11). Therefore, a random-effects model was used. The pooled estimate favored the VR intervention for trait anxiety scores (MD −7.72, 95% CI −13.72 to −1.71; *P*=.01; Figure 4).

#### Depressive Symptoms

Five studies assessed depressive symptoms [36,45,47,49,50]. Because different measurement tools were used, meta-analysis was performed only for 2 studies [49,50] that used the Center for Epidemiologic Studies Depression Scale (CES-D). The other 3 studies used the Beck Depression Inventory (BDI) [36], PROMIS Depression Short Form 8a [47], and the Hamilton Depression Rating Scale (HAMD) [45], respectively. These 3 studies were not included in the quantitative synthesis and were instead summarized narratively, with all reporting lower depression-related scores in the VR intervention groups than in the control groups. The 2 studies that used the CES-D were pooled using the MD as the effect measure [49,50]. The heterogeneity test showed low heterogeneity across studies (*I*²=33%; *P*=.22). Therefore, a fixed-effect model was used. The meta-analysis of the 2 CES-D studies showed lower depressive symptom scores in the VR intervention group than in the usual care group (MD −3.48, 95% CI −6.77 to −0.18; *P*=.04; Figure 4) [49,50]. However, the model-based sensitivity analysis showed that the interpretation of this outcome changed after switching to the random-effects model, indicating that the finding was dependent on model choice. Therefore, although the primary analysis suggested a possible beneficial effect, the effect of VR interventions on depressive symptoms should be interpreted cautiously rather than as definitive evidence of benefit.

### Effects of VR-Based Exercise on Physical Function

This study performed meta-analyses for the TUG, gait speed, STS-5, STS-10, STS-60, and 6MWT.

#### TUG

Two studies [41,49] reported TUG results. The MD was used as the pooled effect measure. The heterogeneity test showed no obvious heterogeneity across studies (*I*²=0%; *P*=.92). Therefore, a fixed-effect model was used. The pooled results showed that, compared with usual care, the VR intervention group had a 1.28-second shorter TUG completion time. However, the difference was not statistically significant (MD −1.28, 95% CI −2.73 to 0.18; *P*=.09; Figure 5). Because fewer than 10 studies were included for this outcome, funnel plots and statistical tests for publication bias were not performed.

**Figure 5.**
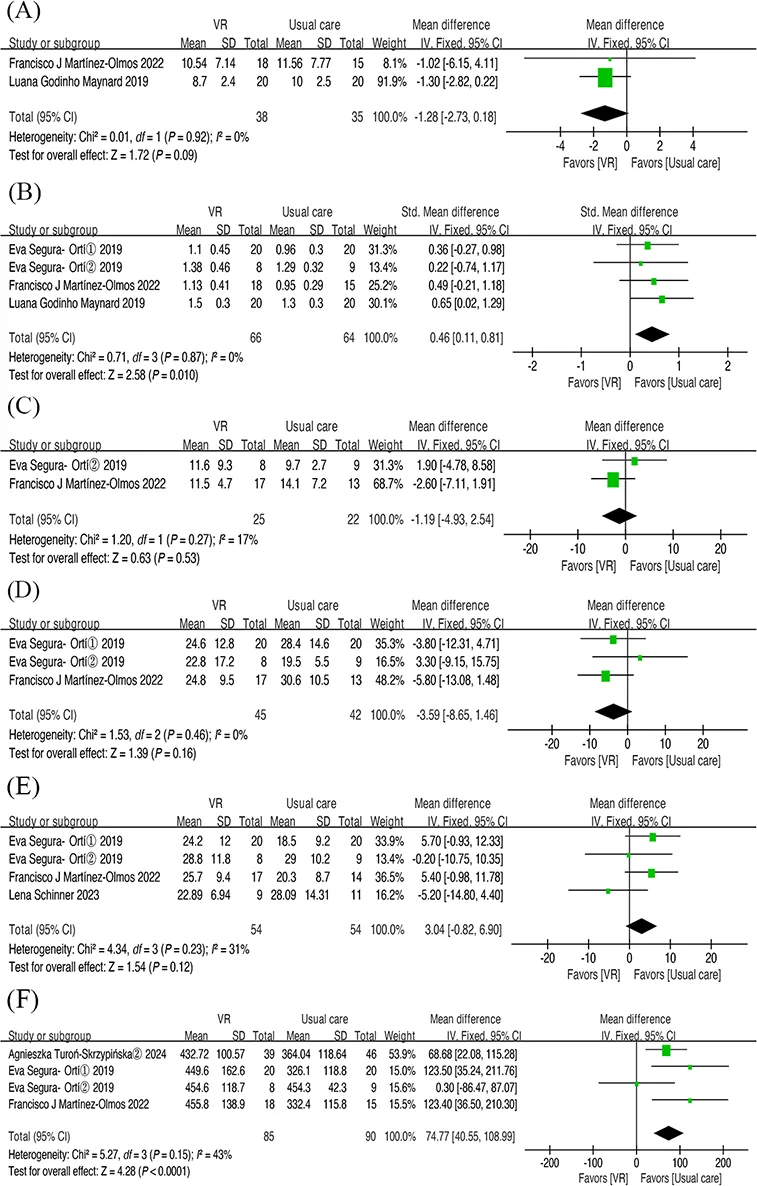
Forest plots of the effects of virtual reality (VR) interventions on physical function. (A) Forest plot of the effect of VR interventions on the timed up and go test [41,49]. (B) Forest plot of the effect of VR interventions on gait speed [41-43,49]. (C) Forest plot of the effect of VR interventions on the 5-times Sit-to-Stand test [41,43]. (D) Forest plot of the effect of VR interventions on the 10-times Sit-to-Stand test [41-43]. (E) Forest plot of the effect of VR interventions on the 1-minute Sit-to-Stand test [41-43,48]. (F) Forest plot of the effect of VR interventions on the 6-Minute Walk Test [37,41-43].

#### Gait Speed

Four studies [41-43,49] reported gait speed. Two studies [41,43] used the 4-meter gait speed test, and 2 studies [42,49] used the 10-meter gait speed test. Therefore, the SMD was used as the pooled effect measure. The heterogeneity test showed no obvious heterogeneity across studies (*I*²=0%; *P*=.87). A fixed-effect model was used. The pooled results showed that gait speed was higher in the VR intervention group than in the usual care group, with a statistically significant difference (SMD 0.46, 95% CI 0.11 to 0.81; *P*=.01; Figure 5). Sensitivity analysis was performed using the leave-one-out method. After each individual study was excluded in turn, the direction of the pooled effect did not change. However, statistical significance was unstable in some analyses (*P*=.009-.08; the sensitivity analysis forest plot is provided in Figure S3 in Multimedia Appendix 5).

#### STS-5

Two studies [41,43] reported STS-5 results. The MD was used as the pooled effect measure. The heterogeneity test showed low heterogeneity across studies (*I*²=17%; *P*=.27). Therefore, a fixed-effect model was used. The pooled results showed no statistically significant difference in STS-5 completion time between the VR intervention and usual care groups (MD −1.19, 95% CI −4.93 to 2.54; *P*=.53; Figure 5).

#### STS-10

Three studies [41-43] reported STS-10 results. The MD was used as the pooled effect measure. The heterogeneity test showed no obvious heterogeneity across studies (*I*²=0%; *P*=.46). Therefore, a fixed-effect model was used. The pooled results showed no statistically significant difference in STS-10 completion time between the VR intervention and usual care groups (MD −3.59, 95% CI −8.65 to 1.46; *P*=.16; Figure 5). Sensitivity analysis was performed using the leave-one-out method. After each individual study was excluded in turn, the direction of the pooled effect did not change, and the results remained statistically nonsignificant (*P*=.08-.67; the sensitivity analysis forest plot is provided in Figure S4 in Multimedia Appendix 5).

#### STS-60

Four studies [41-43,48] reported STS-60 results. The MD was used as the pooled effect measure. The heterogeneity test showed low heterogeneity across studies (*I*²=31%; *P*=.23). Therefore, a fixed-effect model was used. The pooled results showed no statistically significant difference in the number of STS-60 repetitions between the VR intervention and usual care groups (MD 3.04, 95% CI −0.82 to 6.90; *P*=.12; Figure 5). Sensitivity analysis showed that, after excluding the study by Schinner et al [48], heterogeneity disappeared among the remaining 3 studies (*I*²=0%; *P*=.62) [41-43]. The pooled estimate favored the VR intervention after excluding this study (MD 4.63, 95% CI 0.41-8.84; *P*=.03). Excluding the other individual studies did not substantially change the pooled result. Because the number of included studies was limited, no further subgroup analysis was conducted to explore the source of heterogeneity (the sensitivity analysis forest plot is provided in Figure S5 in Multimedia Appendix 5).

#### 6MWT

Four studies [37,41-43] reported 6MWT results. The MD was used as the pooled effect measure. The heterogeneity test showed mild-to-moderate heterogeneity across studies (*I*²=43%; *P*=.15). Therefore, a fixed-effect model was used. The pooled results showed that, compared with usual care, VR interventions significantly increased the 6-minute walk distance (MD 74.77, 95% CI 40.55-108.99; *P*<.001; Figure 5). Sensitivity analysis was performed using the leave-one-out method. After each individual study was excluded in turn, the direction of the pooled effect did not change, and all results remained statistically significant (all *P*<.001). This suggests that the pooled result for this outcome was relatively stable (the sensitivity analysis forest plot is provided in Figure S6 in Multimedia Appendix 5).

### Effects of VR-Based Exercise on Vital Signs

This study performed meta-analyses for HR, RR, SBP, DBP, and SpO₂.

#### HR

Three studies [23,25,49] reported HR results. The MD was used as the pooled effect measure. The heterogeneity test showed no obvious heterogeneity across studies (*I*²=0%; *P*=.82). Therefore, a fixed-effect model was used. The pooled estimate suggested lower HR in the VR intervention group than in the usual care group (MD −3.64, 95% CI −6.96 to −0.31; *P*=.03; Figure 6). Sensitivity analysis was performed using the leave-one-out method. After excluding the study by Elzeky et al [25], the pooled effect was no longer statistically significant (*P*=.35). This finding suggests that the HR outcome was sensitive to a single study and that the stability of the pooled result was limited (the sensitivity analysis forest plot is provided in Figure S7 in Multimedia Appendix 5).

**Figure 6.**
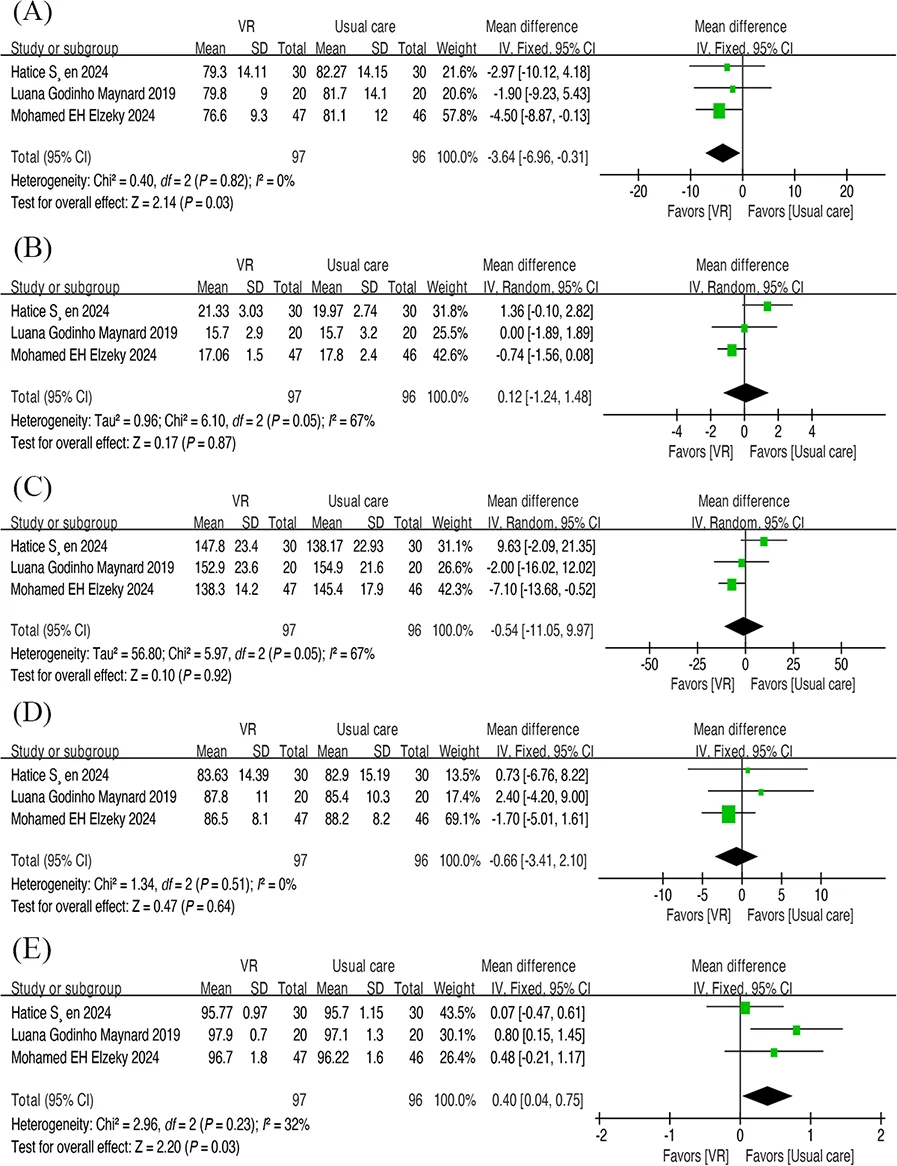
Forest plots of the effects of virtual reality (VR) interventions on vital signs. (A) Forest plot of the effect of VR interventions on heart rate [23,25,49]. (B) Forest plot of the effect of VR interventions on respiratory rate [23,25,49]. (C) Forest plot of the effect of VR interventions on systolic blood pressure [23,25,49]. (D) Forest plot of the effect of VR interventions on diastolic blood pressure [23,25,49]. (E) Forest plot of the effect of VR interventions on peripheral oxygen saturation [23,25,49].

#### RR

Three studies [23,25,49] reported RR results. The MD was used as the pooled effect measure. The heterogeneity test showed moderate heterogeneity across studies (*I*²=67%; *P*=.05). Therefore, a random-effects model was used. The pooled results showed no statistically significant difference in RR between the VR intervention and usual care groups (MD 0.12, 95% CI −1.24 to 1.48; *P*=.87; Figure 6). Sensitivity analysis was performed using the leave-one-out method. After any single study was excluded, the pooled result remained statistically nonsignificant (all *P*>.05). This finding was consistent with the primary analysis and suggests that current evidence does not show a clear effect of VR interventions on RR (the sensitivity analysis forest plot is provided in Figure S8 in Multimedia Appendix 5).

#### SBS

Three studies [23,25,49] reported SBP results. The MD was used as the pooled effect measure. The heterogeneity test showed moderate heterogeneity across studies (*I*²=67%; *P*=.05). Therefore, a random-effects model was used. The pooled results showed no statistically significant difference in SBP between the VR intervention and usual care groups (MD −0.54, 95% CI −11.05 to 9.97; *P*=.92; Figure 6). Sensitivity analysis using the leave-one-out method showed that, after excluding the study by Şen et al [23], heterogeneity disappeared among the remaining 2 studies [25,49] (*I*²=0%; *P*=.52), and the pooled estimate suggested a lower SBP in the VR intervention group than in the usual care group (MD −6.18, 95% CI −12.13 to −0.23; *P*=.04). This finding suggests that the SBP outcome was sensitive to a single study and that the stability of the pooled result was limited (the sensitivity analysis forest plot is provided in Figure S9 in Multimedia Appendix 5).

#### DBP

Three studies [23,25,49] reported DBP results. The MD was used as the pooled effect measure. The heterogeneity test showed no obvious heterogeneity across studies (*I*²=0%; *P*=.51). Therefore, a fixed-effect model was used. The pooled results showed no statistically significant difference in DBP between the VR intervention and usual care groups (MD −0.66, 95% CI −3.41 to 2.10; *P*=.64; Figure 6). Sensitivity analysis was performed using the leave-one-out method. After any single study was excluded, the pooled result remained statistically nonsignificant (all *P*>.05). This finding was consistent with the primary analysis and suggests that current evidence does not show a clear effect of VR interventions on DBP (the sensitivity analysis forest plot is provided in Figure S10 in Multimedia Appendix 5).

#### SpO_2_

Three studies [23,25,49] reported SpO₂ results. The MD was used as the pooled effect measure. The heterogeneity test showed low heterogeneity across studies (*I*²=32%; *P*=.23). Therefore, a fixed-effect model was used. The pooled estimate suggested a possible slight increase in SpO₂ in the VR intervention group compared with the usual care group (MD 0.40, 95% CI 0.04 to 0.75; *P*=.03; Figure 6). However, the leave-one-out sensitivity analysis showed that after excluding the study by Maynard et al [49], the pooled effect was no longer statistically significant (*P*=.30), suggesting that the pooled estimate was also sensitive to the inclusion of specific studies (the sensitivity analysis forest plot is provided in Figure S11 in Multimedia Appendix 5). In addition, the model-based sensitivity analysis showed that the pooled result for SpO2 was sensitive to model choice. The random-effects model showed a nonsignificant result (MD 0.42, 95% CI −0.02 to 0.85; *P*=.06), indicating that the statistical significance of this outcome was not robust across models. Therefore, although the primary analysis suggested a possible increase in SpO2, evidence for this outcome remains uncertain and should be interpreted cautiously.

### Effects of VR-Based Exercise on Health-Related Quality of Life

#### Overview

This study performed meta-analyses for 8 dimensions of health-related quality of life: physical functioning (PF), role-physical (RP), bodily pain (BP), general health (GH), vitality (VT), social functioning (SF), role-emotional (RE), and mental health (MH).

#### PF

PF reflects the ability to perform daily physical activities. Higher scores indicate better PF. Three studies [42,44,49] reported PF results. The MD was used as the pooled effect measure. The heterogeneity test showed no obvious heterogeneity across studies (*I*²=0%; *P*=.59). Therefore, a fixed-effect model was used. The pooled estimate favored the VR intervention for PF scores (MD 11.68, 95% CI 5.38-17.98; *P*<.001; Figure 7). Sensitivity analysis was performed using the leave-one-out method. After excluding the study by Feng et al [44], the pooled effect was no longer statistically significant (*P*=.20). This finding suggests that the PF outcome was sensitive to a single study and that the stability of the pooled result was limited (the sensitivity analysis forest plot is provided in Figure S12 in Multimedia Appendix 5).

**Figure 7.**
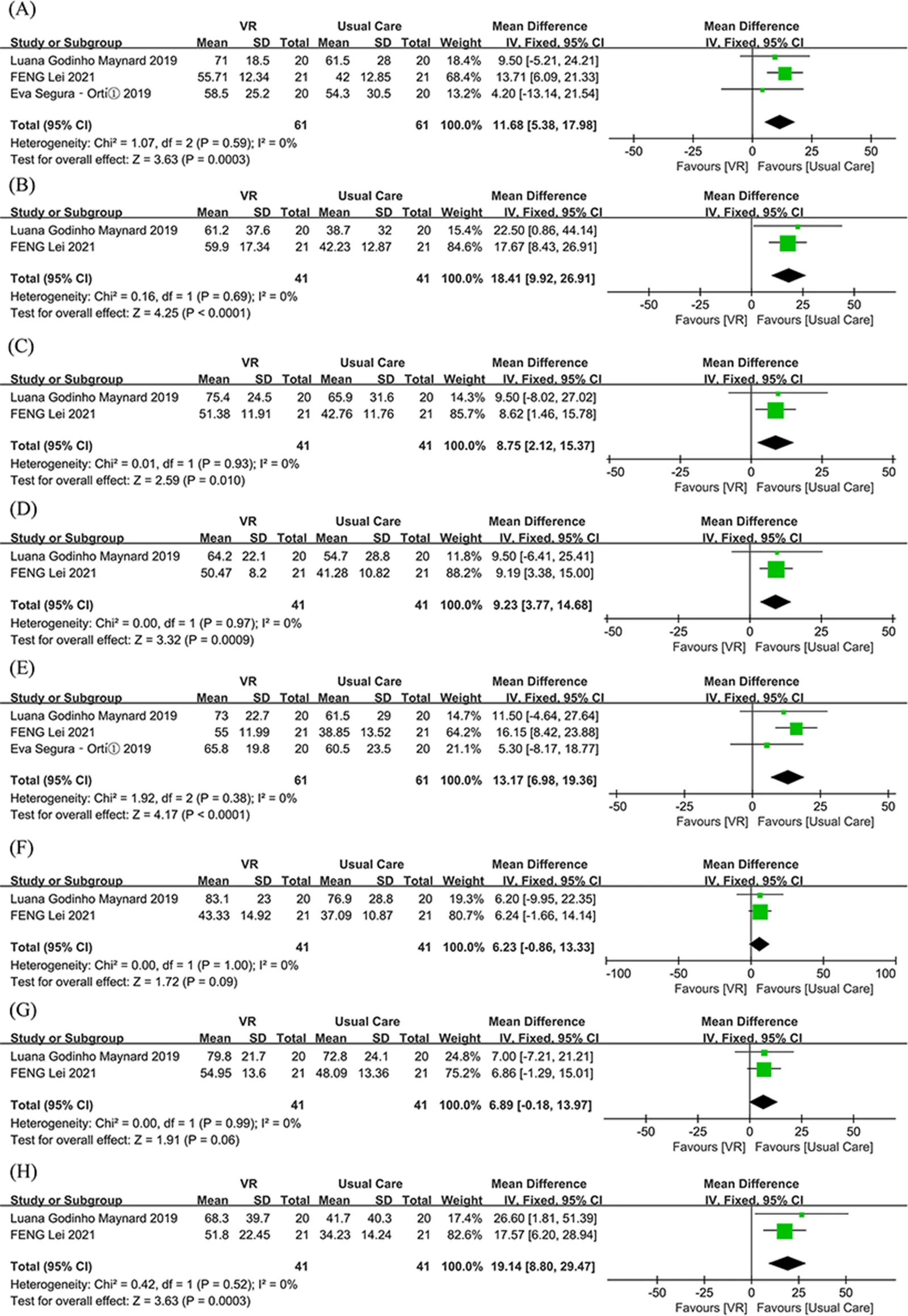
Forest plots of the effects of virtual reality (VR) interventions on health-related quality of life. (A) Forest plot of the effect of VR interventions on physical functioning [42,44,49]. (B) Forest plot of the effect of VR interventions on role-physical [44,49]. (C) Forest plot of the effect of VR interventions on bodily pain [44,49]. (D) Forest plot of the effect of VR interventions on general health [44,49]. (E) Forest plot of the effect of VR interventions on vitality [42,44,49]. (F) Forest plot of the effect of VR interventions on social functioning [44,49]. (G) Forest plot of the effect of VR interventions on role emotional [44,49]. (H) Forest plot of the effect of VR interventions on mental health [44,49].

#### RP

RP reflects limitations in daily role activities due to physical health problems. Higher scores indicate fewer role limitations. Two studies [44,49] reported RP results. The MD was used as the pooled effect measure. The heterogeneity test showed no obvious heterogeneity across studies (*I*²=0%; *P*=.69). Therefore, a fixed-effect model was used. The pooled estimate favored the VR intervention for RP scores (MD 18.41, 95% CI 9.92-26.91; *P*<.001; Figure 7).

#### BP

BP reflects the effect of BP on daily activities and work ability. Higher scores indicate less pain-related interference. Two studies [44,49] reported BP results. The MD was used as the pooled effect measure. The heterogeneity test showed no obvious heterogeneity across studies (*I*²=0%; *P*=.93). Therefore, a fixed-effect model was used. The pooled estimate favored the VR intervention for BP scores (MD 8.75, 95% CI 2.12-15.37; *P*=.01; Figure 7).

#### GH

GH reflects subjective perceptions of overall health and future health expectations. Higher scores indicate better perceived health. Two studies [44,49] reported GH results. The MD was used as the pooled effect measure. The heterogeneity test showed no obvious heterogeneity across studies (*I*²=0%; *P*=.97). Therefore, a fixed-effect model was used. The pooled estimate favored the VR intervention for GH scores (MD 9.23, 95% CI 3.77 to 14.68; *P*<.001; Figure 7).

#### VT

VT reflects energy level and fatigue. Higher scores indicate better VT. Three studies [42,44,49] reported VT results. The MD was used as the pooled effect measure. The heterogeneity test showed no obvious heterogeneity across studies (*I*²=0%; *P*=.38). Therefore, a fixed-effect model was used. The pooled estimate favored the VR intervention for VT scores (MD 13.17, 95% CI 6.98-19.36; *P*<.001; Figure 7). Sensitivity analysis was performed using the leave-one-out method. After excluding the study by Feng et al [44], the pooled effect was no longer statistically significant (*P*=.14). This finding suggests that the VT outcome was sensitive to a single study and that the stability of the pooled result was limited (the sensitivity analysis forest plot is provided in Figure S13 in Multimedia Appendix 5).

#### SF

SF reflects the effect of physical or emotional problems on social activities. Higher scores indicate better SF. Two studies [44,49] reported SF results. The MD was used as the pooled effect measure. The heterogeneity test showed no obvious heterogeneity across studies (*I*²=0%; *P*>.99). Therefore, a fixed-effect model was used. The pooled results showed no statistically significant difference in SF scores between the VR intervention and usual care groups (MD 6.23, 95% CI −0.86 to 13.33; *P*=.09; Figure 7).

#### RE

RE reflects limitations in daily role activities due to emotional problems. Higher scores indicate better emotional role functioning. Two studies [44,49] reported RE results. The MD was used as the pooled effect measure. The heterogeneity test showed no obvious heterogeneity across studies (*I*²=0%; *P*=.99). Therefore, a fixed-effect model was used. The pooled results showed no statistically significant difference in RE scores between the VR intervention and usual care groups (MD 6.89, 95% CI −0.18 to 13.97; *P*=.06; Figure 7).

#### MH

MH reflects psychological status and emotional stability. Higher scores indicate better MH. Two studies [44,49] reported MH results. The MD was used as the pooled effect measure. The heterogeneity test showed no obvious heterogeneity across studies (*I*²=0%; *P*=.52). Therefore, a fixed-effect model was used. The pooled estimate favored the VR intervention for MH scores (MD 19.14, 95% CI 8.80 to 29.47; *P*<.001; Figure 7).

### Sensitivity Analyses and Additional Analyses Subgroup

Sensitivity analyses were performed for outcomes with at least 3 studies using a leave-one-out approach. These analyses assessed whether the pooled effect estimates were substantially influenced by any single study. The results of sensitivity analyses are reported in the corresponding outcome subsections, and the related forest plots are provided in Multimedia Appendix 3. Because several physical function outcomes were mainly informed by studies from the Segura-Ortí research team, we performed an exploratory leave-team-out sensitivity assessment (the sensitivity analysis forest plot is provided in Multimedia Appendix 6).

Formal subgroup analyses were not conducted. Although subgroup analyses were considered according to clinically relevant factors, including VR immersion level, application scenario, comparator type, and intervention duration, the number of studies available for each pooled outcome was limited. Most outcomes included only 2 to 4 studies, which was insufficient to support reliable subgroup comparisons. Meta-regression was also not conducted for the same reason.

## Discussion

### Principal Findings

This systematic review and meta-analysis evaluated whether VR-based interventions were more effective than usual care in improving symptom management–related outcomes in patients undergoing hemodialysis. Using GRADEpro, we assessed the certainty of evidence for each outcome. Overall, the findings suggest that the effects of VR are scenario-specific rather than universal.

- Procedural pain distraction during arteriovenous fistula cannulation: the strongest evidence was observed for procedural pain distraction during arteriovenous fistula cannulation. Moderate-certainty evidence showed that VR-based distraction reduced cannulation pain compared with usual care, supporting the use of VR in this specific procedural setting.
- Exercise-supported rehabilitation and selected physical function outcomes: moderate-certainty evidence showed that VR-supported exercise improved gait speed and 6-minute walk distance and reduced depressive symptoms. These findings suggest that VR-assisted exercise may benefit selected physical and psychological outcomes, although intervention formats varied across studies.
- Uncertain outcomes, quality of life, and safety-related indicators: evidence for anxiety, STS-60, and several health-related quality-of-life outcomes was less certain. Low-certainty evidence suggested possible benefits in selected quality-of-life domains, but these findings should be interpreted cautiously. Changes in vital signs should be regarded primarily as indicators of safety and tolerability rather than core efficacy outcomes.

### Pain

This study showed that VR interventions reduced arteriovenous fistula cannulation pain in patients undergoing hemodialysis compared with usual care. This finding is generally consistent with a recent systematic review and meta-analysis [51], which suggested that VR can relieve procedure-related pain in hemodialysis. Current evidence is mainly concentrated in the setting of arteriovenous fistula cannulation. Compared with traditional distraction methods, such as music, guided imagery, or conventional videos, VR may provide a stronger sense of presence and greater multisensory engagement within a short period. This makes it more suitable for procedural pain during arteriovenous fistula cannulation, where the intervention window is clear and the duration is short. However, current evidence mainly comes from comparisons between VR and usual care. It remains unclear whether VR provides additional benefits over other active nonpharmacological interventions [20]. Future studies should distinguish pain during a single cannulation episode from the cumulative pain experience caused by repeated cannulation in patients undergoing long-term hemodialysis, such as pain anticipation, procedure-related anxiety, and pain memory. Future research should also assess implementation-related outcomes, including vascular access safety, device-related discomfort, willingness to reuse VR, workload for health care professionals, and cost-effectiveness. These data are needed to determine whether VR can move beyond a short-term analgesic tool and become a sustainable symptom management strategy in clinical practice.

### Physical Function

For physical function, improvements with VR interventions were mainly observed in functional walking outcomes, such as gait speed and the 6MWT. This finding is consistent with previous reviews of VR-based exercise training and studies of intradialytic exercise, which suggest that intradialytic exercise is more likely to improve sustained activity capacity and lower-limb functional performance [29,31]. Most VR training programs in the included studies were nonimmersive gamified exercise interventions. Patients completed tasks through repeated lower-limb movements and received immediate feedback and difficulty adjustment. This format may improve exercise engagement and training continuity. Notably, sensitivity analysis for STS-60 showed that this outcome was sensitive to a single study. After excluding the study by Schinner et al [48], heterogeneity decreased and the pooled effect became statistically significant. This may be because that study used neuromuscular electrical stimulation as the main intervention, while VR was used only as a distraction or supportive experience. It was not centered on gamified active VR exercise. Compared with other VR exercise studies, it differed in training mechanism, exercise load, and degree of active patient participation. This clinical heterogeneity may affect the stability of the pooled effect. The STS-60 result should therefore be interpreted with caution. In addition, the clinical value of VR is not to replace conventional exercise rehabilitation. Rather, it may provide a more feasible way to support intradialytic exercise. Its advantage lies in embedding low- to moderate-intensity activity into the dialysis process. Contextualized tasks, immediate feedback, and difficulty adjustment may help patients sustain participation. For older, frail, disabled, or cognitively impaired patients undergoing hemodialysis, VR-assisted exercise should not use a uniform intensity or fixed format. It should be implemented according to fall risk, functional reserve, cognitive understanding, hemodynamic stability, and vascular access safety.

### Psychological Symptoms

For psychological symptoms, the pooled estimates suggested potential effects of VR-based interventions on trait anxiety and depressive symptoms, but these findings should be interpreted cautiously. State anxiety and trait anxiety reflect different psychological dimensions. State anxiety is more dependent on the measurement time point and the immediate dialysis context. If the scale is assessed before cannulation, after cannulation, during dialysis, or after the intervention, the direction and magnitude of the effect may differ substantially. Improvements in depressive symptoms may partly reflect exercise participation, supervision, increased engagement, emotion regulation, or self-efficacy, rather than a direct psychotherapeutic effect of VR itself. Because the trait-anxiety result was based on only 2 studies, it should be regarded as exploratory; whether short-term VR interventions can change relatively stable anxiety tendencies remains uncertain [31]. Therefore, current evidence is more suitable for supporting the adjunctive role of VR in improving dialysis-related negative emotional experiences, rather than proving a definite psychotherapeutic effect.

### Quality of Life

For health-related quality of life, this study observed improvements in only selected dimensions. The findings are insufficient to support a broad effect of VR on overall quality of life. Improvements were mainly found in PF, BP, GH, VT, and MH. These dimensions may be more closely related to dialysis experience, exercise participation, and subjective health perception. In contrast, SF and RE dimensions are more strongly influenced by family support, social roles, disease burden, and the long-term care environment. Short-term VR interventions may be insufficient to produce stable changes in these domains [52]. Previous reviews suggested that VR training may improve psychological health, social participation, and self-efficacy. However, these effects do not necessarily translate into stable improvements in the SF or RE dimensions of the 36-item Short Form Health Survey (SF-36). Therefore, the role of VR in improving quality of life should be understood as adjunctive support for selected symptoms and functional experiences. It should not be interpreted as a short-term comprehensive intervention that reshapes the broader social life of patients.

### Other Outcomes

For objective indicators, this study showed that the effects of VR on vital signs were unstable. HR, blood pressure, and SpO2 in patients undergoing hemodialysis are easily affected by volume status, ultrafiltration volume, dialysis prescription, measurement time point, and related medications. Therefore, short-term changes in vital signs should not be directly interpreted as stable physiological effects of VR. They are more appropriate as auxiliary indicators of safety and tolerability. Narrative evidence further suggests that VR may have potential effects on sleep, happiness, mindfulness, fatigue, physical activity, and selected physiological or metabolic indicators. One study [46] showed that VR combined with aromatherapy may be superior to VR alone in improving sleep quality and happiness. This may be related to the joint involvement of visual immersion and olfactory stimulation in emotion regulation. However, this result came from a single study. Whether the combined intervention has an independent added benefit still requires further validation. The 2 interventions may act through different input pathways to the limbic system, and may jointly activate the limbic system, and regulate autonomic tone. However, this conclusion came from a small factorial study, and the specific incremental effect of the combined intervention still needs independent confirmation. Some studies also showed that VR natural scenes may improve mindfulness and fatigue by directing attention, reducing negative rumination, and enhancing relaxation. However, the effect size and durability remain unclear [53]. In terms of physical function, narrative evidence showed that VR exercise or somatosensory games may improve physical activity level and exercise self-efficacy, reduce fall risk, and have positive effects on local muscle strength and handgrip strength [42,44,48]. These findings are consistent with the meta-analysis results showing improvements in gait speed and the 6MWT. Together, they suggest that the functional benefits of VR-related exercise interventions may be concentrated in lower-limb activity, walking endurance, and daily activity participation. In contrast, evidence for handgrip strength and body composition remains insufficient. Short-term interventions may also be unable to produce substantial changes in muscle mass or fat mass.

Studies by Turoń-Skrzypińska and colleagues [36-39] found that VR exercise may reduce inflammatory and bone metabolism–related markers, including IL-1β, IL-6, IL-8, TNF-α, PCSK9, and SOST. Regular exercise itself can exert physiological effects through anti-inflammatory, lipid metabolism, and bone metabolism pathways [54-56]. Therefore, these changes may be closely related to the exercise component and should not be simply attributed to VR technology itself. Given that these findings mainly came from the same research team, and that all interventions combined VR with active exercise, they are better regarded as mechanistic hypotheses rather than definitive conclusions at this stage. Future studies should use standardized VR protocols, active comparator designs, and consistent core outcomes. This will help distinguish the contributions of VR presentation, exercise training, and their interaction to symptom experience, functional capacity, and inflammatory or metabolic indicators.

### Adverse Events

In terms of safety, current evidence does not suggest a serious safety signal for VR in patients undergoing hemodialysis. Previous reviews have extracted adverse events as part of data collection. However, safety reporting in the included studies was inconsistent. Some studies reported no adverse events. Some reported adverse events without specifying the type. Others did not report safety outcomes. Based on the original trials included in this review [26,46], VR-related discomfort was mainly mild and manageable. Reported events included eye pain, device-related discomfort, removal of VR glasses during the intervention, and muscle cramps related to exercise or neuromuscular electrical stimulation. Because most studies lacked a uniform definition of adverse events and an active monitoring process, the current findings are more suitable for supporting the generally acceptable short-term tolerability of VR. They are insufficient to prove its long-term safety. Future studies should incorporate proactive and standardized safety monitoring for VR-related discomfort, including dizziness, nausea, visual fatigue, eye pain, and headache, as well as hemodynamic fluctuations, vascular access–related events, device cleaning and disinfection, and reasons for intervention discontinuation. Because these discomfort symptoms may overlap with common intradialytic symptoms, passive adverse-event reporting alone may underestimate tolerability issues [57].

### Digital Health Implementation Considerations

For integration into routine hemodialysis care, VR should be considered a workflow-enabled digital intervention rather than a stand-alone device [58]. Dialysis units should establish local protocols for patient suitability screening, brief orientation, supervised first exposure, stop criteria, and feasibility and acceptability documentation [59]. During cannulation or intradialytic use, device positioning should preserve visibility of the vascular access site, dialysis lines, patient responses, and machine alarms; VR should be paused whenever access assessment, nursing procedures, or clinical instability requires direct observation [60]. To improve tolerability, centers may use short sessions, visually stable low-motion content, avoidance of rapid navigation or excessive dynamic scenes, and active symptom checks, especially for patients with frailty, sensory or cognitive limitations, intradialytic instability, or low digital literacy [58,61,62]. Future research may explore adaptive content design, real-time symptom monitoring, and emerging mitigation strategies, such as neuromodulation-based approaches for reducing VR-related motion sickness, although further safety and feasibility evaluation is needed before use in hemodialysis settings [63]. Staff training should cover device setup, troubleshooting, cleaning and disinfection, safety monitoring, and adverse event documentation, with clear responsibility for storage, maintenance, technical support, and cost tracking. Future pragmatic trials should evaluate reach, adoption, fidelity, feasibility, acceptability, equity, resource use, and sustainability alongside clinical outcomes.

### Strengths and Limitations

This study has several strengths. First, this review synthesized evidence from 19 RCTs involving 1228 adult patients undergoing hemodialysis across 10 countries. The study sources covered different regions and countries with different income levels. This enhanced the population diversity and clinical generalizability of the evidence to some extent. Second, unlike previous studies that mainly focused on a single VR application scenario, this study included both VR distraction for cannulation pain and VR training during hemodialysis. This provides a more comprehensive picture of the application spectrum of VR in hemodialysis symptom management. Finally, this study further integrated multidimensional outcomes, including pain, psychological symptoms, physical function, health-related quality of life, vital signs, nutritional and inflammatory metabolic indicators, adverse events, and patient experience. This helps evaluate the clinical translation value of VR from the perspectives of effectiveness, safety, and implementation feasibility. Based on this evidence, this study not only updates the evidence on VR in hemodialysis but also further distinguishes relatively stable benefit scenarios from outcomes that remain uncertain. These findings provide a clearer basis for future intervention design, core outcome selection, and optimization of digital symptom management strategies in dialysis units.

This study has several limitations. First, the evidence base was limited. Several outcomes, including psychological symptoms, TUG, and selected health-related quality-of-life domains, were informed by only a few studies, which restricted subgroup analyses and heterogeneity exploration and increased the influence of individual studies on pooled estimates. The robustness of some pooled findings was also limited: state anxiety, depressive symptoms, and SpO2 were sensitive to model choice, and the 6MWT result was sensitive to the exclusion of individual studies in leave-one-out analyses. These findings should therefore be interpreted cautiously. In addition, evidence for several physical function outcomes was largely derived from studies conducted by the same research team or research program. Although potential participant overlap was assessed and duplicate extraction of participants or outcomes was avoided, residual center- or team-dominant effects cannot be excluded. Fewer than 10 studies were available for each outcome, precluding formal assessment of publication bias. Second, the methodological quality of the included studies was limited. Most trials were small and single-center, and reporting of allocation concealment and blinding was often insufficient. These issues may partly reflect the practical challenges of blinding and standardizing VR interventions, including device type, immersion level, and intervention content. Third, substantial clinical and methodological heterogeneity existed across studies. VR interventions varied in type and application scenario, including immersive distraction during cannulation, gamified exercise during hemodialysis, home-based somatosensory exercise, psychological support, and multicomponent interventions. Differences in intervention dose, comparator conditions, and assessment time points further limited the interpretability of pooled estimates. Therefore, the effects of VR should be interpreted according to specific clinical contexts and outcomes. Fourth, adverse events and tolerability outcomes were insufficiently and inconsistently reported. Although no major safety concerns were identified, the limited reporting of dizziness, visual fatigue, nausea, discomfort, device-related problems, and dialysis-related safety issues prevents firm conclusions about the safety of VR interventions in hemodialysis settings. Finally, in studies combining VR with exercise, aromatherapy, or other cointerventions, the independent effect of VR could not be clearly separated from the effects of accompanying interventions, attention, or other non-VR components. Future trials should use rigorous designs, larger and more diverse samples, standardized reporting, and appropriate comparator groups to better estimate the independent and additive effects of VR.

### Conclusions

The findings of this systematic review and meta-analysis suggest that VR should not be interpreted as a single homogeneous intervention for symptom management in adults undergoing hemodialysis. Instead, the current evidence supports a scenario-specific interpretation. Brief VR-based distraction may reduce procedural pain during arteriovenous fistula cannulation, whereas VR-supported exercise or rehabilitation programs may improve selected physical function outcomes, such as gait speed and 6MWT, and promote participation in intradialytic exercise. Evidence for depressive symptoms, selected dimensions of health-related quality of life, treatment experience, and multicomponent adjunctive interventions remains less certain. These findings should be interpreted cautiously because the included studies varied substantially in VR type, application scenario, intervention dose, comparator condition, and outcome measurement. In studies combining VR with exercise or other cointerventions, the independent effect of VR could not be clearly separated from that of the accompanying intervention. In addition, the evidence base was limited by small study numbers, short follow-up, low certainty of evidence, and limited reporting of safety and tolerability outcomes. Future trials should distinguish procedural distraction, VR-supported exercise rehabilitation, and multicomponent adjunctive interventions, and should further evaluate the effectiveness, safety, feasibility, and implementation of VR in routine hemodialysis care.

## Supplementary material

10.2196/102331 Multimedia Appendix 1 Detailed search strategies for all databases.

10.2196/102331 Multimedia Appendix 2 Studies excluded from the meta-analysis and narratively summarized studies or outcomes.

10.2196/102331 Multimedia Appendix 3 Grading of Recommendations Assessment, Development and Evaluation evidence profile and certainty assessment for outcomes included in the quantitative meta-analysis.

10.2196/102331 Multimedia Appendix 4 Sensitivity analysis comparing random-effect and fixed-effect models for outcomes included in the meta-analysis.

10.2196/102331 Multimedia Appendix 5 Sensitivity analysis forest plots for outcomes included in the meta-analysis.

10.2196/102331 Multimedia Appendix 6 Sensitivity analyses excluding studies by the Segura-Ortí team for physical function outcomes.

10.2196/102331 Checklist 1 PRISMA 2020 checklist.

## Abbreviations

6MWT: 6-Minute Walk Test
BDI: Beck Depression Inventory
BP: bodily pain
CES-D: Center for Epidemiologic Studies Depression Scale
CKD: chronic kidney disease
DBP: diastolic blood pressure
GAD-7: Generalized Anxiety Disorder-7
GH: general health
GRADE: Grading of Recommendations Assessment, Development and Evaluation
HAMA: Hamilton Anxiety Rating Scale
HAMD: Hamilton Depression Rating Scale
HMD: head-mounted display
HR: heart rate
MD: mean difference
MH: mental health
PF: physical functioning
PRISMA: Preferred Reporting Items for Systematic Reviews and Meta-Analyses
RCT: randomized controlled trial
RE: role-emotional
RoB 2: Cochrane risk of bias 2 tool for randomized trials
RP: role-physical
RR: respiratory rate
SBP: systolic blood pressure
SF: social functioning
SF-36: 36-item Short Form Health Survey
SMD: standardized mean difference
SOST: sclerostin
SpO2: peripheral oxygen saturation
STAI: State-Trait Anxiety Inventory
STAI-S: State-Trait Anxiety Inventory–State
STAI-T: State-Trait Anxiety Inventory–Trial
STS-10: 10-times Sit-to-Stand test
STS-5: 5-times Sit-to-Stand test
STS-60: 1-minute Sit-to-Stand test
TUG: Timed Up and Go test
VAS: Visual Analogue Scale
VR: virtual reality
VT: vitality
WHA: World Health Assembly
