# Supplementary material for: Effectiveness and Safety of Virtual Reality Interventions for Symptom Management in Adults Undergoing Hemodialysis: Systematic Review and Meta-Analysis of Randomized Controlled Trials
Source: J Med Internet Res. 2026 Sep 28;28:e102331. doi: 10.2196/102331 (PMC13618410; doi:10.2196/102331)
Supplement: Supplementary file 1 [file jmir-v28-e102331-s001.docx]

| **Databases** | **Set#** | **Searched for** | **Results** | **Search Time** |
| --- | --- | --- | --- | --- |
| **CINAHL Complete** | S1 | SU("Renal Replacement Therapy") OR XB("Replacement Therapy, Kidney" OR "Kidney Replacement Therapies" OR "Replacement Therapies, Kidney" OR "Therapies, Kidney Replacement" OR "Therapy, Kidney Replacement" OR "Kidney Replacement Therapy" OR "Replacement Therapy, Renal" OR "Renal Replacement Therapies" OR "Replacement Therapies, Renal" OR "Therapies, Renal Replacement" OR "Therapy, Renal Replacement" OR haemodialysis OR kidney failure OR end-stage renal failure OR end-stage kidney disease OR renal dialys* OR hemodialys* OR maintenance hemodialys* OR end-stage renal disease OR chronic kidney disease OR blood purification OR "Kidney Replacement Therapies" OR "Replacement Therapies, Kidney" OR "Replacement Therapies, Renal" OR "Therapies, Kidney Replacement" OR "Therapy, Kidney Replacement" OR "Replacement Therapy, Kidney" OR "Kidney Replacement Therapy" OR "Therapies, Renal Replacement" OR "Therapy, Renal Replacement" OR "Renal Replacement Therapies" OR dialysis therapy OR dialysis treatment OR kidney dialysis OR kidney support OR renal support) | 53949 | 2026-01-06 |
|  | S2 | SU("Virtual Reality") OR XB("Virtual Reality Immersion Therapy" OR "Virtual Reality Therapy" OR virtual environment OR virtual task OR virtual world OR serious gam* OR virtual gam* OR video gam* OR computer gam* OR exergam* OR digital technology OR VR OR immersi* OR head mounted display OR head-mounted display OR htc vive OR Nintendo OR Kinect OR Wii OR computer simulation OR user-computer interface OR virtual patient OR virtual hospital OR virtual system OR virtual setting OR virtual simulation OR virtual ward OR computer stimulat* OR simulated environment OR artificial reality OR augmented reality OR patient stimulation OR gaming OR 3D stimulation OR 3D game OR 3D setting OR 3D environment OR 3D gaming OR "Educational Virtual Realities" OR educational virtual realities OR "Instructional Virtual Reality" OR "Instructional Virtual Realities" OR "Educational Virtual Reality") | 29664 |  |
|  | S3 | S2 AND S1 | 50 |  |
| **Embase** | #1 | 'renal replacement therapy'/exp | 284577 | 2026-01-06 |
|  | #2 | 'replacement therapy, renal':ti,kw,ab OR haemodialysis:ti,kw,ab OR kidney failure:ti,kw,ab OR end-stage renal failure:ti,kw,ab OR end-stage kidney disease:ti,kw,ab OR renal dialys*:ti,kw,ab OR hemodialys*:ti,kw,ab OR maintenance hemodialys*:ti,kw,ab OR end-stage renal disease:ti,kw,ab OR chronic kidney disease:ti,kw,ab OR blood purification:ti,kw,ab OR kidney replacement therapies:ti,kw,ab OR replacement therapies, kidney:ti,kw,ab OR replacement therapies, renal:ti,kw,ab OR therapies, kidney replacement:ti,kw,ab OR therapy, kidney replacement:ti,kw,ab OR replacement therapy, kidney:ti,kw,ab OR kidney replacement therapy:ti,kw,ab OR therapies, renal replacement:ti,kw,ab OR therapy, renal replacement:ti,kw,ab OR renal replacement therapies:ti,kw,ab OR dialysis therapy:ti,kw,ab OR dialysis treatment:ti,kw,ab OR kidney dialysis:ti,kw,ab OR kidney support:ti,kw,ab OR renal support:ti,kw,ab | 376951 |  |
|  | #3 | 'virtual reality'/exp | 39726 |  |
|  | #4 | 'virtual reality immersion therapy':ti,kw,ab OR 'virtual reality therapy':ti,kw,ab OR virtual environment:ti,kw,ab OR virtual task:ti,kw,ab OR virtual world:ti,kw,ab OR serious gam*:ti,kw,ab OR virtual gam*:ti,kw,ab OR video gam*:ti,kw,ab OR computer gam*:ti,kw,ab OR exergam*:ti,kw,ab OR digital technology:ti,kw,ab OR vr:ti,kw,ab OR immersi*:ti,kw,ab OR head mounted display:ti,kw,ab OR head-mounted display:ti,kw,ab OR htc vive:ti,kw,ab OR nintendo:ti,kw,ab OR kinect:ti,kw,ab OR wii:ti,kw,ab OR computer simulation:ti,kw,ab OR user-computer interface:ti,kw,ab OR virtual patient:ti,kw,ab OR virtual hospital:ti,kw,ab OR virtual system:ti,kw,ab OR virtual setting:ti,kw,ab OR virtual simulation:ti,kw,ab OR virtual ward:ti,kw,ab OR computer stimulat*:ti,kw,ab OR simulated environment:ti,kw,ab OR artificial reality:ti,kw,ab OR augmented reality:ti,kw,ab OR patient stimulation:ti,kw,ab OR gaming:ti,kw,ab OR 3d stimulation:ti,kw,ab OR 3d game:ti,kw,ab OR 3d setting:ti,kw,ab OR 3d environment:ti,kw,ab OR 3d gaming:ti,kw,ab OR educational virtual realities:ti,kw,ab OR instructional virtual reality:ti,kw,ab OR instructional virtual realities:ti,kw,ab OR educational virtual reality:ti,kw,ab | 135334 |  |
|  | #5 | #1 OR #2 | 497930 |  |
|  | #6 | #3 OR #4 | 155156 |  |
|  | #7 | #5 AND #6 | 587 |  |
| **PsycINFO** | S1 | SU("Renal Replacement Therapy") OR AB("Replacement Therapy, Kidney" OR "Kidney Replacement Therapies" OR "Replacement Therapies, Kidney" OR "Therapies, Kidney Replacement" OR "Therapy, Kidney Replacement" OR "Kidney Replacement Therapy" OR "Replacement Therapy, Renal" OR "Renal Replacement Therapies" OR "Replacement Therapies, Renal" OR "Therapies, Renal Replacement" OR "Therapy, Renal Replacement" OR haemodialysis OR kidney failure OR end-stage renal failure OR end-stage kidney disease OR renal dialys* OR hemodialys* OR maintenance hemodialys* OR end-stage renal disease OR chronic kidney disease OR blood purification OR "Kidney Replacement Therapies" OR "Replacement Therapies, Kidney" OR "Replacement Therapies, Renal" OR "Therapies, Kidney Replacement" OR "Therapy, Kidney Replacement" OR "Replacement Therapy, Kidney" OR "Kidney Replacement Therapy" OR "Therapies, Renal Replacement" OR "Therapy, Renal Replacement" OR "Renal Replacement Therapies" OR dialysis therapy OR dialysis treatment OR kidney dialysis OR kidney support OR renal support) | 4247 | 2026-01-06  2026-01-06  2026-01-06 |
|  | S2 | SU("Virtual Reality") OR AB("Virtual Reality Immersion Therapy" OR "Virtual Reality Therapy" OR virtual environment OR virtual task OR virtual world OR serious gam* OR virtual gam* OR video gam* OR computer gam* OR exergam* OR digital technology OR VR OR immersi* OR head mounted display OR head-mounted display OR htc vive OR Nintendo OR Kinect OR Wii OR computer simulation OR user-computer interface OR virtual patient OR virtual hospital OR virtual system OR virtual setting OR virtual simulation OR virtual ward OR computer stimulat* OR simulated environment OR artificial reality OR augmented reality OR patient stimulation OR gaming OR 3D stimulation OR 3D game OR 3D setting OR 3D environment OR 3D gaming OR "Educational Virtual Realities" OR educational virtual realities OR "Instructional Virtual Reality" OR "Instructional Virtual Realities" OR "Educational Virtual Reality") | 49503 |  |
|  | S3 | S2 AND S1 | 18 |  |
| **PubMed** | 1 | "Renal Replacement Therapy"[Mesh] | 247483 | 2026-01-05 |
|  | 2 | "Replacement Therapy, Kidney"[Title/Abstract] OR "Kidney Replacement Therapies"[Title/Abstract] OR "Replacement Therapies, Kidney"[Title/Abstract] OR "Therapies, Kidney Replacement"[Title/Abstract] OR "Therapy, Kidney Replacement"[Title/Abstract] OR "Kidney Replacement Therapy"[Title/Abstract] OR "Replacement Therapy, Renal"[Title/Abstract] OR "Renal Replacement Therapies"[Title/Abstract] OR "Replacement Therapies, Renal"[Title/Abstract] OR "Therapies, Renal Replacement"[Title/Abstract] OR "Therapy, Renal Replacement"[Title/Abstract] OR haemodialysis[Title/Abstract] OR kidney failure[Title/Abstract] OR end-stage renal failure[Title/Abstract] OR end-stage kidney disease[Title/Abstract] OR renal dialys*[Title/Abstract] OR hemodialys*[Title/Abstract] OR maintenance hemodialys*[Title/Abstract] OR end-stage renal disease[Title/Abstract] OR chronic kidney disease[Title/Abstract] OR blood purification[Title/Abstract] OR "Kidney Replacement Therapies"[Title/Abstract] OR "Replacement Therapies, Kidney"[Title/Abstract] OR "Replacement Therapies, Renal"[Title/Abstract] OR "Therapies, Kidney Replacement"[Title/Abstract] OR "Therapy, Kidney Replacement"[Title/Abstract] OR "Replacement Therapy, Kidney"[Title/Abstract] OR "Kidney Replacement Therapy"[Title/Abstract] OR "Therapies, Renal Replacement"[Title/Abstract] OR "Therapy, Renal Replacement"[Title/Abstract] OR "Renal Replacement Therapies"[Title/Abstract] OR dialysis therapy[Title/Abstract] OR dialysis treatment[Title/Abstract] OR kidney dialysis[Title/Abstract] OR kidney support[Title/Abstract] OR renal support[Title/Abstract] | 232580 | 2026-01-05 |
|  | 3 | "Virtual Reality"[Mesh] | 9810 | 2026-01-05 |
|  | 4 | "Virtual Reality Immersion Therapy"[Title/Abstract] OR "Virtual Reality Therapy"[Title/Abstract] OR virtual environment[Title/Abstract] OR virtual task[Title/Abstract] OR virtual world[Title/Abstract] OR serious gam*[Title/Abstract] OR virtual gam*[Title/Abstract] OR video gam*[Title/Abstract] OR computer gam*[Title/Abstract] OR exergam*[Title/Abstract] OR digital technology[Title/Abstract] OR VR[Title/Abstract] OR immersi*[Title/Abstract] OR head mounted display[Title/Abstract] OR head-mounted display[Title/Abstract] OR htc vive[Title/Abstract] OR Nintendo[Title/Abstract] OR Kinect[Title/Abstract] OR Wii[Title/Abstract] OR computer simulation[Title/Abstract] OR user-computer interface[Title/Abstract] OR virtual patient[Title/Abstract] OR virtual hospital[Title/Abstract] OR virtual system[Title/Abstract] OR virtual setting[Title/Abstract] OR virtual simulation[Title/Abstract] OR virtual ward[Title/Abstract] OR computer stimulat*[Title/Abstract] OR simulated environment[Title/Abstract] OR artificial reality[Title/Abstract] OR augmented reality[Title/Abstract] OR patient stimulation[Title/Abstract] OR gaming[Title/Abstract] OR 3D stimulation[Title/Abstract] OR 3D game[Title/Abstract] OR 3D setting[Title/Abstract] OR 3D environment[Title/Abstract] OR 3D gaming[Title/Abstract] OR "Educational Virtual Realities"[Title/Abstract] OR educational virtual realities[Title/Abstract] OR "Instructional Virtual Reality"[Title/Abstract] OR "Instructional Virtual Realities"[Title/Abstract] OR "Educational Virtual Reality"[Title/Abstract] | 110060 | 2026-01-05 |
|  | 5 | ("Renal Replacement Therapy"[Mesh]) OR ("Replacement Therapy, Kidney"[Title/Abstract] OR "Kidney Replacement Therapies"[Title/Abstract] OR "Replacement Therapies, Kidney"[Title/Abstract] OR "Therapies, Kidney Replacement"[Title/Abstract] OR "Therapy, Kidney Replacement"[Title/Abstract] OR "Kidney Replacement Therapy"[Title/Abstract] OR "Replacement Therapy, Renal"[Title/Abstract] OR "Renal Replacement Therapies"[Title/Abstract] OR "Replacement Therapies, Renal"[Title/Abstract] OR "Therapies, Renal Replacement"[Title/Abstract] OR "Therapy, Renal Replacement"[Title/Abstract] OR haemodialysis[Title/Abstract] OR kidney failure[Title/Abstract] OR end-stage renal failure[Title/Abstract] OR end-stage kidney disease[Title/Abstract] OR renal dialys*[Title/Abstract] OR hemodialys*[Title/Abstract] OR maintenance hemodialys*[Title/Abstract] OR end-stage renal disease[Title/Abstract] OR chronic kidney disease[Title/Abstract] OR blood purification[Title/Abstract] OR "Kidney Replacement Therapies"[Title/Abstract] OR "Replacement Therapies, Kidney"[Title/Abstract] OR "Replacement Therapies, Renal"[Title/Abstract] OR "Therapies, Kidney Replacement"[Title/Abstract] OR "Therapy, Kidney Replacement"[Title/Abstract] OR "Replacement Therapy, Kidney"[Title/Abstract] OR "Kidney Replacement Therapy"[Title/Abstract] OR "Therapies, Renal Replacement"[Title/Abstract] OR "Therapy, Renal Replacement"[Title/Abstract] OR "Renal Replacement Therapies"[Title/Abstract] OR dialysis therapy[Title/Abstract] OR dialysis treatment[Title/Abstract] OR kidney dialysis[Title/Abstract] OR kidney support[Title/Abstract] OR renal support[Title/Abstract]) | 384172 | 2026-01-05 |
|  | 6 | ("Virtual Reality"[Mesh]) OR ("Virtual Reality Immersion Therapy"[Title/Abstract] OR "Virtual Reality Therapy"[Title/Abstract] OR virtual environment[Title/Abstract] OR virtual task[Title/Abstract] OR virtual world[Title/Abstract] OR serious gam*[Title/Abstract] OR virtual gam*[Title/Abstract] OR video gam*[Title/Abstract] OR computer gam*[Title/Abstract] OR exergam*[Title/Abstract] OR digital technology[Title/Abstract] OR VR[Title/Abstract] OR immersi*[Title/Abstract] OR head mounted display[Title/Abstract] OR head-mounted display[Title/Abstract] OR htc vive[Title/Abstract] OR Nintendo[Title/Abstract] OR Kinect[Title/Abstract] OR Wii[Title/Abstract] OR computer simulation[Title/Abstract] OR user-computer interface[Title/Abstract] OR virtual patient[Title/Abstract] OR virtual hospital[Title/Abstract] OR virtual system[Title/Abstract] OR virtual setting[Title/Abstract] OR virtual simulation[Title/Abstract] OR virtual ward[Title/Abstract] OR computer stimulat*[Title/Abstract] OR simulated environment[Title/Abstract] OR artificial reality[Title/Abstract] OR augmented reality[Title/Abstract] OR patient stimulation[Title/Abstract] OR gaming[Title/Abstract] OR 3D stimulation[Title/Abstract] OR 3D game[Title/Abstract] OR 3D setting[Title/Abstract] OR 3D environment[Title/Abstract] OR 3D gaming[Title/Abstract] OR "Educational Virtual Realities"[Title/Abstract] OR educational virtual realities[Title/Abstract] OR "Instructional Virtual Reality"[Title/Abstract] OR "Instructional Virtual Realities"[Title/Abstract] OR "Educational Virtual Reality"[Title/Abstract]) | 112979 | 2026-01-05 |
|  | 7 | (("Virtual Reality"[Mesh]) OR ("Virtual Reality Immersion Therapy"[Title/Abstract] OR "Virtual Reality Therapy"[Title/Abstract] OR virtual environment[Title/Abstract] OR virtual task[Title/Abstract] OR virtual world[Title/Abstract] OR serious gam*[Title/Abstract] OR virtual gam*[Title/Abstract] OR video gam*[Title/Abstract] OR computer gam*[Title/Abstract] OR exergam*[Title/Abstract] OR digital technology[Title/Abstract] OR VR[Title/Abstract] OR immersi*[Title/Abstract] OR head mounted display[Title/Abstract] OR head-mounted display[Title/Abstract] OR htc vive[Title/Abstract] OR Nintendo[Title/Abstract] OR Kinect[Title/Abstract] OR Wii[Title/Abstract] OR computer simulation[Title/Abstract] OR user-computer interface[Title/Abstract] OR virtual patient[Title/Abstract] OR virtual hospital[Title/Abstract] OR virtual system[Title/Abstract] OR virtual setting[Title/Abstract] OR virtual simulation[Title/Abstract] OR virtual ward[Title/Abstract] OR computer stimulat*[Title/Abstract] OR simulated environment[Title/Abstract] OR artificial reality[Title/Abstract] OR augmented reality[Title/Abstract] OR patient stimulation[Title/Abstract] OR gaming[Title/Abstract] OR 3D stimulation[Title/Abstract] OR 3D game[Title/Abstract] OR 3D setting[Title/Abstract] OR 3D environment[Title/Abstract] OR 3D gaming[Title/Abstract] OR "Educational Virtual Realities"[Title/Abstract] OR educational virtual realities[Title/Abstract] OR "Instructional Virtual Reality"[Title/Abstract] OR "Instructional Virtual Realities"[Title/Abstract] OR "Educational Virtual Reality"[Title/Abstract])) AND (("Renal Replacement Therapy"[Mesh]) OR ("Replacement Therapy, Kidney"[Title/Abstract] OR "Kidney Replacement Therapies"[Title/Abstract] OR "Replacement Therapies, Kidney"[Title/Abstract] OR "Therapies, Kidney Replacement"[Title/Abstract] OR "Therapy, Kidney Replacement"[Title/Abstract] OR "Kidney Replacement Therapy"[Title/Abstract] OR "Replacement Therapy, Renal"[Title/Abstract] OR "Renal Replacement Therapies"[Title/Abstract] OR "Replacement Therapies, Renal"[Title/Abstract] OR "Therapies, Renal Replacement"[Title/Abstract] OR "Therapy, Renal Replacement"[Title/Abstract] OR haemodialysis[Title/Abstract] OR kidney failure[Title/Abstract] OR end-stage renal failure[Title/Abstract] OR end-stage kidney disease[Title/Abstract] OR renal dialys*[Title/Abstract] OR hemodialys*[Title/Abstract] OR maintenance hemodialys*[Title/Abstract] OR end-stage renal disease[Title/Abstract] OR chronic kidney disease[Title/Abstract] OR blood purification[Title/Abstract] OR "Kidney Replacement Therapies"[Title/Abstract] OR "Replacement Therapies, Kidney"[Title/Abstract] OR "Replacement Therapies, Renal"[Title/Abstract] OR "Therapies, Kidney Replacement"[Title/Abstract] OR "Therapy, Kidney Replacement"[Title/Abstract] OR "Replacement Therapy, Kidney"[Title/Abstract] OR "Kidney Replacement Therapy"[Title/Abstract] OR "Therapies, Renal Replacement"[Title/Abstract] OR "Therapy, Renal Replacement"[Title/Abstract] OR "Renal Replacement Therapies"[Title/Abstract] OR dialysis therapy[Title/Abstract] OR dialysis treatment[Title/Abstract] OR kidney dialysis[Title/Abstract] OR kidney support[Title/Abstract] OR renal support[Title/Abstract])) | 325 | 2026-01-05 |
| **Web of Science** | 1 | kidney replacement therap* OR renal replacement therap* OR hemodialys* OR haemodialys* OR maintenance hemodialys* OR dialys* OR dialysis therap* OR dialysis treatment OR renal dialys* OR kidney dialys* OR kidney failure OR renal failure OR end-stage kidney disease OR end stage kidney disease OR ESKD OR end-stage renal disease OR end stage renal disease OR ESRD OR end-stage renal failure OR end stage renal failure OR chronic kidney disease OR CKD OR blood purification OR renal support OR kidney support (Topic) | 454820 | 2026-01-06 |
|  | 2 | virtual reality OR VR OR virtual reality therap* OR virtual reality immersion therap* OR immersive virtual reality OR augmented reality OR artificial reality OR virtual environment* OR virtual world* OR virtual simulation* OR simulated environment* OR computer simulation OR head mounted display* OR head-mounted display* OR serious gam* OR virtual gam* OR video gam* OR computer gam* OR exergam* OR gaming OR 3D game* OR 3D environment* OR 3D gaming OR Kinect OR Wii OR Nintendo OR HTC Vive OR user-computer interface OR virtual patient* OR virtual hospital* OR (immers* NEAR/3 (virtual reality OR VR)) (Topic) | 619726 |  |
|  | 3 | #2 AND #1 | 460 |  |
| **Ovid MEDLINE(R) ALL** | 1 | Virtual Reality/ | 9515 | 2026-01-02 |
|  | 2 | (Virtual Reality Immersion Therapy OR Virtual Reality Therapy OR virtual environment OR virtual task OR virtual world OR serious gam* OR virtual gam* OR video gam* OR computer gam* OR exergam* OR digital technology OR VR OR immersi* OR head mounted display OR head-mounted display OR htc vive OR Nintendo OR Kinect OR Wii OR computer simulation OR user-computer interface OR virtual patient OR virtual hospital OR virtual system OR virtual setting OR virtual simulation OR virtual ward OR computer stimulat* OR simulated environment OR artificial reality OR augmented reality OR patient stimulation OR gaming OR 3D stimulation OR 3D game OR 3D setting OR 3D environment OR 3D gaming OR Educational Virtual Realities OR educational virtual realities OR Instructional Virtual Reality OR Instructional Virtual Realities OR Educational Virtual Reality).ab,ti,kw. | 109185 |  |
|  | 3 | Renal Replacement Therapy/ | 7641 |  |
|  | 4 | (Replacement Therapy, Kidney OR Kidney Replacement Therapies OR Replacement Therapies, Kidney OR Therapies, Kidney Replacement OR Therapy, Kidney Replacement OR Kidney Replacement Therapy OR Replacement Therapy, Renal OR Renal Replacement Therapies OR Replacement Therapies, Renal OR Therapies, Renal Replacement OR Therapy, Renal Replacement OR haemodialysis OR kidney failure OR end-stage renal failure OR end-stage kidney disease OR renal dialys* OR hemodialys* OR maintenance hemodialys* OR end-stage renal disease OR chronic kidney disease OR blood purification OR Kidney Replacement Therapies OR Replacement Therapies, Kidney OR Replacement Therapies, Renal OR Therapies, Kidney Replacement OR Therapy, Kidney Replacement OR Replacement Therapy, Kidney OR Kidney Replacement Therapy OR Therapies, Renal Replacement OR Therapy, Renal Replacement OR Renal Replacement Therapies OR dialysis therapy OR dialysis treatment OR kidney dialysis OR kidney support OR renal support).ab,ti,kw. | 230753 |  |
|  | 5 | 1 OR 2 | 111843 |  |
|  | 6 | 3 OR 4 | 234963 |  |
|  | 7 | 5 AND 6 | 220 |  |
| **Scopus** | 1 | (ALL("Replacement Therapy, Kidney") OR ALL("Kidney Replacement Therapies") OR ALL("Replacement Therapies, Kidney") OR ALL("Therapies, Kidney Replacement") OR ALL("Therapy, Kidney Replacement") OR ALL("Kidney Replacement Therapy") OR ALL("Replacement Therapy, Renal") OR ALL("Renal Replacement Therapies") OR ALL("Replacement Therapies, Renal") OR ALL("Therapies, Renal Replacement") OR ALL("Therapy, Renal Replacement") OR ALL(haemodialysis) OR ALL(kidney failure) OR ALL(end-stage renal failure) OR ALL(end-stage kidney disease) OR ALL(renal dialys*) OR ALL(hemodialys*) OR ALL(maintenance hemodialys*) OR ALL(end-stage renal disease) OR ALL(chronic kidney disease) OR ALL(blood purification) OR ALL("Kidney Replacement Therapies") OR ALL("Replacement Therapies, Kidney") OR ALL("Replacement Therapies, Renal") OR ALL("Therapies, Kidney Replacement") OR ALL("Therapy, Kidney Replacement") OR ALL("Replacement Therapy, Kidney") OR ALL("Kidney Replacement Therapy") OR ALL("Therapies, Renal Replacement") OR ALL("Therapy, Renal Replacement") OR ALL("Renal Replacement Therapies") OR ALL(dialysis therapy) OR ALL(dialysis treatment) OR ALL(kidney dialysis) OR ALL(kidney support) OR ALL(renal support)) AND (ALL("Virtual Reality Immersion Therapy") OR ALL("Virtual Reality Therapy") OR ALL(virtual environment) OR ALL(virtual task) OR ALL(virtual world) OR ALL(serious gam*) OR ALL(virtual gam*) OR ALL(video gam*) OR ALL(computer gam*) OR ALL(exergam*) OR ALL(digital technology) OR ALL(VR) OR ALL(immersi*) OR ALL(head mounted display) OR ALL(head-mounted display) OR ALL(htc vive) OR ALL(Nintendo) OR ALL(Kinect) OR ALL(Wii) OR ALL(computer simulation) OR ALL(user-computer interface) OR ALL(virtual patient) OR ALL(virtual hospital) OR ALL(virtual system) OR ALL(virtual setting) OR ALL(virtual simulation) OR ALL(virtual ward) OR ALL(computer stimulat*) OR ALL(simulated environment) OR ALL(artificial reality) OR ALL(augmented reality) OR ALL(patient stimulation) OR ALL(gaming) OR ALL(3D stimulation) OR ALL(3D game) OR ALL(3D setting) OR ALL(3D environment) OR ALL(3D gaming) OR ALL("Educational Virtual Realities") OR ALL(educational virtual realities) OR ALL("Instructional Virtual Reality") OR ALL("Instructional Virtual Realities") OR ALL("Educational Virtual Reality")) | — |  |
| **Cochrane Library** | #1 | MeSH descriptor: [Renal Replacement Therapy] explode all trees | 12883 | 2026-01-06  2026-01-06 |
|  | #2 | ('Replacement Therapy, Kidney' OR 'Kidney Replacement Therapies' OR 'Replacement Therapies, Kidney' OR 'Therapies, Kidney Replacement' OR 'Therapy, Kidney Replacement' OR 'Kidney Replacement Therapy' OR 'Replacement Therapy, Renal' OR 'Renal Replacement Therapies' OR 'Replacement Therapies, Renal' OR 'Therapies, Renal Replacement' OR 'Therapy, Renal Replacement' OR 'haemodialysis' OR 'kidney failure' OR 'end-stage renal failure' OR 'end-stage kidney disease' OR 'renal dialys*' OR 'hemodialys*' OR 'maintenance hemodialys*' OR 'end-stage renal disease' OR 'chronic kidney disease' OR 'blood purification' OR ' Kidney Replacement Therapies' OR ' Replacement Therapies, Kidney' OR ' Replacement Therapies, Renal' OR ' Therapies, Kidney Replacement' OR ' Therapy, Kidney Replacement' OR ' Replacement Therapy, Kidney' OR ' Kidney Replacement Therapy' OR ' Therapies, Renal Replacement' OR ' Therapy, Renal Replacement' OR ' Renal Replacement Therapies' OR 'dialysis therapy' OR 'dialysis treatment' OR 'kidney dialysis' OR 'kidney support' OR 'renal support'):ti,kw,ab | 55847 |  |
|  | #3 | MeSH descriptor: [Virtual Reality] explode all trees | 1675 |  |
|  | #4 | ('Virtual Reality Immersion Therapy' OR 'Virtual Reality Therapy' OR 'virtual environment' OR 'virtual task' OR 'virtual world' OR 'serious gam*' OR 'virtual gam*' OR 'video gam*' OR 'computer gam*' OR 'exergam*' OR 'digital technology' OR 'VR' OR 'immersi*' OR 'head mounted display' OR 'head-mounted display' OR 'htc vive' OR 'Nintendo' OR 'Kinect' OR 'Wii' OR 'computer simulation' OR 'user-computer interface' OR 'virtual patient ' OR 'virtual hospital ' OR 'virtual system' OR 'virtual setting' OR 'virtual simulation' OR 'virtual ward' OR 'computer stimulat* ' OR 'simulated environment' OR 'artificial reality' OR 'augmented reality' OR 'patient stimulation' OR 'gaming' OR '3D stimulation' OR '3D game' OR '3D setting' OR '3D environment' OR '3D gaming' OR ' Educational Virtual Realities' OR 'educational virtual realities' OR ' Instructional Virtual Reality' OR ' Instructional Virtual Realities' OR ' Educational Virtual Reality'):ti,kw,ab | 49641 |  |
|  | #5 | #1 OR #2 | 59474 |  |
|  | #6 | #3 OR #4 | 49699 |  |
|  | #7 | #5 AND #6 | 700 |  |
| **Proquest** | S1 | mainsubject("Virtual Reality Immersion Therapy") OR summary("Virtual Reality Therapy" OR "virtual environment" OR "virtual task" OR "virtual world" OR serious gam* OR virtual gam* OR video gam* OR computer gam* OR exergam* OR "digital technology" OR VR OR immersi* OR "head mounted display" OR "head-mounted display" OR "htc vive" OR "Nintendo" OR "Kinect" OR "Wii" OR "computer simulation" OR "user-computer interface" OR "virtual patient" OR "virtual hospital" OR "virtual system" OR "virtual setting" OR "virtual simulation" OR "virtual ward" OR computer stimulat* OR "simulated environment" OR "artificial reality" OR "augmented reality" OR "patient stimulation" OR gaming OR "3D stimulation" OR "3D game" OR "3D setting" OR "3D environment" OR "3D gaming" OR "Educational Virtual Realities" OR "educational virtual realities" OR "Instructional Virtual Reality" OR "Instructional Virtual Realities" OR "Educational Virtual Reality") | 1652446 | 2026-01-06 |
|  | S2 | mainsubject("Replacement Therapy, Kidney") OR summary("Kidney Replacement Therapies" OR "Replacement Therapies, Kidney" OR "Therapies, Kidney Replacement" OR "Therapy, Kidney Replacement" OR "Kidney Replacement Therapy" OR "Replacement Therapy, Renal" OR "Renal Replacement Therapies" OR "Replacement Therapies, Renal" OR "Therapies, Renal Replacement" OR "Therapy, Renal Replacement" OR haemodialysis OR "kidney failure" OR "end-stage renal failure" OR "end-stage kidney disease" OR renal dialys* OR hemodialys* OR "maintenance hemodialys*" OR "end-stage renal disease" OR "chronic kidney disease" OR "blood purification" OR "dialysis therapy" OR "dialysis treatment" OR "kidney dialysis" OR "kidney support" OR "renal support") | 389439 |  |
|  | S3 | [S1] AND [S2] | 511 |  |
| **CNKI** | 1 | （篇关摘：肾透析+ 血液透析 + 透析 + 血液透析滤过 + 血液滤过 + 超滤 + 血透析 + 维持性血透析 + 终末期肾病 + 血液净化 + 慢性肾病（精确）） AND (篇关摘：虚拟现实 + 虚拟环境 + 虚拟现实暴露疗法 + 虚拟暴露疗法 + 虚拟 + 虚拟游戏 + 电脑虚拟活动 + 电脑虚拟活动 + 互动电子游戏 + 数字疗法 + 沉浸式技术 + 头戴式显示器(精确)) | 119 | 2026-01-06 |
| **WanFang** | 2 | 全部:(肾透析 or 血液透析 or 透析 or 血液透析滤过 or 血液滤过 or 超滤 or 血透析 or 维持性血透析 or 终末期肾病 or 血液净化 or 慢性肾病) and 全部:(虚拟现实 or 虚拟环境 or 虚拟现实暴露疗法 or 虚拟暴露疗法 or 虚拟 or 虚拟游戏 or 电脑虚拟活动 or 电脑虚拟活动 or 互动电子游戏 or 数字疗法 or 沉浸式技术 or 头) and 全部:(随机对照试验 or 随机对照实验 or 随机对照研究) | 400 | 2026-01-06 |
|  |  |  |  |  |
|  |  |  |  |  |
