# Supplementary material for: Effectiveness and Safety of Virtual Reality Interventions for Symptom Management in Adults Undergoing Hemodialysis: Systematic Review and Meta-Analysis of Randomized Controlled Trials
Source: J Med Internet Res. 2026 Sep 28;28:e102331. doi: 10.2196/102331 (PMC13618410; doi:10.2196/102331)
Supplement: Supplementary file 2 [file jmir-v28-e102331-s002.docx]

**Table S1.** Studies fully excluded from the present review and their reasons for exclusion from meta-analysis.

| **Title** | **Study** | **report type** | **Outcome** | **Reason for exclusion from meta-analysis** |
| --- | --- | --- | --- | --- |
| Unravelling the possibilities a cross-over randomised controlled feasibility trial on immersive virtual reality in haemodialysis | Smyth et al., 2024 | Crossover randomized controlled feasibility trial | • Treatment adherence: attendance at scheduled hemodialysis sessions; adherence to fluid allowance • Health-related quality of life: AQoL-6D • Psychological distress: K-5 • Feasibility and acceptability: VR usage; patient feedback; clinician satisfaction • Safety: adverse events | The study used a crossover design, and several outcomes were reported as model-based estimates, test statistics, or narrative feasibility findings rather than period-specific means, standard deviations, or event counts suitable for the planned pairwise meta-analysis. Complete headset usage data were also not available for all participants. |
| Impact of an intradialysis virtual-reality-based exercise program on healthcare resources expenditure: a micro-costing analysis | García-Testal et al., 2022 | Secondary micro-costing analysis of a non-immersive VR-based intradialytic exercise program | • Healthcare resource use: laboratory tests; outpatient visits; radiology tests; medication use; hospital admissions or related healthcare use • Economic outcomes: healthcare resource expenditure; micro-costing indicators | The primary outcomes were healthcare resource consumption and micro-cost indicators, with only 6-minute walking distance recorded at baseline, and this comparison was based on resource use in the 12 months before and after implementation, rather than on eligible VR versus non-VR clinical effect estimates. |
| Understanding how virtual reality forest experience promote physiological and psychological health for patients undergoing hemodialysis | Hsieh and Li, 2022 | Single-group preliminary study of VR forest experience | • Hemodynamic / physiological: HR; HRV • Psychological responses: emotional state questionnaire; qualitative interview data | The study did not include an eligible non-VR control group. Therefore, a between-group effect size comparing VR with usual care, no VR, or another eligible comparator could not be calculated. Some physiological data were also incomplete because of measurement or device-related issues. |
| Positive Psychological Intervention Delivered Using Virtual Reality in Patients on Hemodialysis With Comorbid Depression: Protocol and Design for the Joviality Randomized Controlled Trial | Hernandez et al., 2023 | Randomized controlled trial protocol | Planned outcomes included feasibility, depressive symptoms, psychological well-being, quality of life, treatment adherence, biomarkers, and hospitalization | This was a protocol paper and did not report outcome data. No effect estimates or extractable results were available for meta-analysis. |
| Effect of virtual reality video application on fistula puncture-associated pain in patients undergoing hemodialysis: Assessor-blinded randomized trial | Altınok Ersoy et al., 2024 | Attention controlled and assessor-blinded randomized trial | • Procedural pain: fistula puncture-associated pain measured by VAS at baseline, repeated hemodialysis sessions, and follow-up | The pain VAS scores did not conform to the normal distribution, so the median + interquartile range was used in all cases, and the original mean SD was not used. Transformations are indirect imputations that do not clearly extract means, standard deviations, and single post-intervention endpoints consistent with planned meta-analyses. |
| Effect of intradialytic virtual reality exercise program on lower limb muscle strength in patients on hemodialysis | Reboredo et al., 2024 | Conference abstract of a randomized clinical trial comparing two VR exercise timing protocols | • Lower-limb muscle strength: HF; HA; AE measured by handheld dynamometer | Both groups received the intradialytic non-immersive VR exercise intervention, with the comparison based on exercise timing during the dialysis session rather than VR versus non-VR. |
| The Effect of Intradialytic Exercise Using Virtual Reality on the Body Composition of Patients with Chronic Kidney Disease | Romeu-Perales et al., 2024 | Quasi-experimental before–after substudy of a clinical trial | • Body composition: BMI; FTI; LTI; EIW; PA • Metabolic and nutritional variables: albumin; Hb; potassium; phosphorus; calcium; ferritin; bicarbonate; triglycerides; LDL cholesterol; HDL cholesterol | The study was a quasi-experimental before–after substudy rather than an independent randomized comparison eligible for the planned RCT meta-analysis. The outcomes focused on body composition and nutritional/hydration-related indicators, which were outside the prespecified symptom-management outcomes for quantitative pooling. The study also came from the Segura-Ortí intradialytic VR exercise program, so possible project-level overlap with related reports was considered. |
| Impact of the GoodRENal platform on lower-limbs muscle strength in a Spanish sample | Segura-Ortí et al., 2024 | Conference abstract | • Lower-limb muscle strength: right and left hip flexor strength; right and left hip abductor strength measured by handheld dynamometer | The report was available only as an abstract and provided limited extractable information. Potential overlap with related GoodRENal or Segura-Ortí program reports was also considered, and the available information was insufficient to rule out project- or cohort-level overlap for quantitative pooling. |
| A Pilot Study of Virtual Reality in Hemodialysis for Mitigating Pain and Anxiety: User Experiences and Perceptions | Rodríguez de Galvis et al., 2025 | Prospective, non-randomized, crossover, multicenter pilot study | • Procedural pain: FPS-R • Anxiety: VAS-A • Feasibility and acceptability: VR adherence; number of VR sessions completed; usability; satisfaction; patient perceptions | This study was a non-randomised study, primarily employing a pre-post control design using virtual reality (VR) exposure therapy. Although data on outcome measures such as pain and anxiety were complete and available, the study did not provide a valid randomised between-group comparison and therefore could not be included in the meta-analysis of randomised controlled trials (RCTs). |
| Personalized virtual reality in hemodialysis patients: a multicenter pilot study | Russ et al., 2025 | Multicenter single-group pre–post pilot study | • Psychological response: subjective well-being • Pain: self-reported pain before and after VR exposure • Hemodynamic / physiological: SBP; DBP; HR; SpO₂ • Feasibility and acceptability: treatment tolerance; perceived quality; feasibility of personalized VR | The study used a single-arm pre–post design without a non-VR comparator group. Therefore, a between-group effect size could not be calculated. |
| Effect of a Virtual Reality-Based Exercise Program on Fatigue in Hospitalized Taiwanese End-Stage Renal Disease Patients Undergoing Hemodialysis. | Chou et al., 2018 | Quasi-experimental study | • Fatigue: NFSHD total score and subdomains • Clinical / laboratory parameters: SAlb; BUN; SCr; Hb | The study used a quasi-experimental design rather than random allocation. Although fatigue data were reported, the comparison was not eligible for the RCT-only quantitative synthesis. In addition, the hospitalized setting and short intervention duration differed clinically from most outpatient intradialytic VR studies. |
| Effect of a Virtual Reality Exercise on Patients Undergoing Haemodialysis: A Randomised Controlled Clinical Trial Research Protocol | Meléndez-Oliva et al., 2023 | Randomised controlled clinical trial research protocol | • Functional capacity: balance tests; 4-m walking speed test; STS-5; OLB; TUG; STS-10; STS-60; 6MWT • Psychological status: CAMAE-RD; STAI; BDI-II • Inflammatory markers: IL-6; TNF-α; CRP; ICAM-1; VCAM-1 • Exercise adherence: number of completed exercise sessions • Hemodynamic monitoring: HR; BP before and after exercise sessions | This was a published research protocol and did not report outcome data. Although the planned trial involved random allocation to non-immersive VR exercise versus static pedalling exercise, no effect estimates, means, standard deviations, event counts, or follow-up results. |

Note. AE = ankle extension; AQoL-6D = Assessment of Quality of Life-6D; BDI-II = Beck Depression Inventory-II; BMI = body mass index; BP = blood pressure; BUN = blood urea nitrogen; CAMAE-RD = Multidimensional Questionnaire of Adaptation to the Disease for Renal Patients on Dialysis; CRP = C-reactive protein; DBP = diastolic blood pressure; EIW = extracellular/intracellular water; FPS-R = Faces Pain Scale-Revised; FTI = fat tissue index; HA = hip abduction; Hb = hemoglobin; HDL = high-density lipoprotein; HF = hip flexion; HR = heart rate; HRV = heart rate variability; ICAM-1 = intercellular adhesion molecule-1; IL-6 = interleukin-6; K-5 = Kessler Psychological Distress Scale-5; LDL = low-density lipoprotein; LTI = lean tissue index; NFSHD = Novel Fatigue Scale for Hemodialysis; OLB = one-leg balance test; PA = phase angle; RR = respiratory rate; SAlb = serum albumin; SBP = systolic blood pressure; SCr = serum creatinine; SpO₂ = peripheral oxygen saturation; STAI = State-Trait Anxiety Inventory; STS-5 = five-repetition sit-to-stand test; STS-10 = ten-repetition sit-to-stand test; STS-60 = 60-second sit-to-stand test; TNF-α = tumor necrosis factor-alpha; TUG = Timed Up and Go test; VAS = Visual Analog Scale; VAS-A = Visual Analog Scale for Anxiety; VCAM-1 = vascular cell adhesion molecule-1; VR = virtual reality; 6MWT = Six-Minute Walk Test.

**Table S2.** Included studies or outcomes summarized narratively but not included in quantitative meta-analysis.

| **Title** | **Study** | **Outcome(s) summarized narratively** | **Measurement tool or indicator** | **Reason for not being included in quantitative meta-analysis** |
| --- | --- | --- | --- | --- |
| The effect of virtual reality distraction on haemodialysis patients’ pain and anxiety during arteriovenous fistula puncture a randomised controlled trial | Mohamed EH Elzeky et al., 2024 | • Operation/procedural satisfaction | • Satisfaction visual analogue scale | Satisfaction was a study-specific secondary outcome and was not sufficiently comparable with other prespecified pooled symptom-management outcomes. Therefore, this outcome was summarized narratively rather than quantitatively pooled. |
| A comparative analysis of the impact of virtual reality and rose aroma on anxiety, sleep quality, and happiness among patients undergoing dialysis a randomized control trial with a factorial design | Fahimeh Heirati et al., 2025 | • Sleep quality;  • Happiness | • Pittsburgh Sleep Quality Index (PISQ);  • Oxford Happiness Questionnaire (OHQ) | These outcomes were reported in a factorial trial evaluating VR, aromatherapy, and combined intervention arms. Sleep quality and happiness were not reported with sufficiently comparable measures across other eligible studies, so they were not suitable for meta-analysis. |
| Determination of the Effects of Virtual Reality-BasedVideos on Symptoms, Vital Signs and Comfort inHemodialysis Patients | Dilan Aktepe Coşar et al., 2026 | • Hemodialysis comfort | • Hemodialysis Comfort Scale Version II（HDCS II） | Comfort was measured using a study-specific hemodialysis comfort scale. No sufficiently comparable comfort outcome was available across other studies for quantitative pooling. |
| Effects of Exercise Training Combined with Virtual Reality in Functionality and Health-Related Quality of Life of Patients on Hemodialysis | Luana Godinho Maynard et al., 2019 | • Functional capacity measured by DASI | • Duke Activity Status Index (DASI) | DASI was not reported by other comparable studies and differed from the physical function outcomes used in the quantitative synthesis, such as gait speed, sit-to-stand tests, or 6-minute walk distance. It was therefore summarized narratively. |
| Intradialytic neuromuscular electrical stimulation with optional virtual reality distraction improves not only muscle strength and functional capacity but also serum albumin level in haemodialysis patients a pilot random | Lena Schinner et al., 2023 | • Serum albumin;  • Muscle strength;  • Muscle circumference; • Body composition | • Serum albumin level (g/dl);  • QFM force right (kgf);  • QFM force left (kgf);  • QFM circumference right (cm);  • QFM circumference left (cm);  • Weight (kg);  • BMI (kg/m^2^);  • Fat proportion (%);  • Muscle proportion (%) | These outcomes were either unique to this study or not sufficiently comparable with outcomes reported in other studies. In addition, the intervention involved neuromuscular electrical stimulation with optional VR, and several outcomes had different outcome-specific sample sizes. These factors limited valid quantitative pooling. |
| Intradialytic virtual reality exercise_ Increasing physical activity through technology - sdi.12788 | Eva Segura-Ortí et al., 2019 | • Right-hand grip strength | • Handgrip strength measured by dynamometer | Right-hand grip strength was not pooled with other muscle strength outcomes because the anatomical site, measurement method, and outcome construct differed from lower-limb strength or functional performance outcomes reported in other studies. It was therefore narratively summarized. |
| Virtual Reality Mindfulness and Personalized Exercise for Patients on Hemodialysis with Depressive Symptoms A Feasibility Study | Brett T. Burrows et al., 2023 | • Mindfulness;  • Fatigue | • Five Facet Mindfulness Questionnaire—Short Form (FFMQ-SF);  • SONG-HD Fatigue | These outcomes were reported in a small feasibility study of VR mindfulness plus personalized exercise. Mindfulness and fatigue were not measured consistently across comparable studies, and no suitable pool of studies was available for quantitative synthesis. |
| Virtual reality somatosensory games integrated into the home exercise management and its effects on  fall risk and quality of life in dialysis patients | Feng Lei et al., 2021 | • Exercise self-efficacy; • Fall risk | • Self-Efficacy for Exercise Scale (SEE);  • Morse Fall Scale | Self-efficacy and fall risk were unique outcomes in this study and were not reported in a comparable way by other included studies. These outcomes were therefore summarized narratively rather than quantitatively pooled. |
