# Supplementary material for: Effectiveness and Safety of Virtual Reality Interventions for Symptom Management in Adults Undergoing Hemodialysis: Systematic Review and Meta-Analysis of Randomized Controlled Trials
Source: J Med Internet Res. 2026 Sep 28;28:e102331. doi: 10.2196/102331 (PMC13618410; doi:10.2196/102331)
Supplement: Supplementary file 3 [file jmir-v28-e102331-s003.docx]

| **Certainty assessment** | | | | | | | | **N of patients** | | **Effect** | **Certainty** | **Importance** |
| --- | --- | --- | --- | --- | --- | --- | --- | --- | --- | --- | --- | --- |
| **N of studies** | | **Study design** | **Risk of bias** | **Inconsistency** | **Indirectness** | **Imprecision** | **Other considerations** | **[intervention]** | **[comparison]** | **Absolute (95% CI)** |  |  |
| **VAS（0-10）** | | | | | | | | | | | | |
| 4 | randomised trials | | serious^a^ | not serious | not serious | not serious | none | 147 | 146 | MD **2.44 lower** (2.91 lower to 1.97 lower) | ⨁⨁⨁◯ Moderate^a^ | CRITICAL |
| **State Anxiety** | | | | | | | | | | | | |
| 3 | randomised trials | | serious^a^ | serious^b^ | not serious | serious^d^ | none | 106 | 103 | MD **9.64 lower** (24.43 lower to 5.15 higher) | ⨁◯◯◯ low^a,b,d^ | IMPORTANT |
| **Trait anxiety** | | | | | | | | | | | | |
| 2 | randomised trials | | serious^a^ | serious^b^ | not serious | serious^c^ | none | 59 | 57 | MD **7.72 lower** (13.72 lower to 1.71 lower) | ⨁◯◯◯ Very low^a,b,c^ | IMPORTANT |
| **Depression** | | | | | | | | | | | | |
| 2 | randomised trials | | not serious | not serious | not serious | serious^c^ | none | 57 | 56 | MD **3.48 lower** (6.77 lower to 0.18 lower) | ⨁⨁⨁◯ Moderate^c^ | IMPORTANT |
| **TUG** | | | | | | | | | | | | |
| 2 | randomised trials | | not serious | not serious | not serious | serious^d^ | none | 38 | 35 | MD **1.28 lower** (2.73 lower to 0.18 higher) | ⨁⨁⨁◯ Moderate^d^ | IMPORTANT |
| **Gait speed（m/s)** | | | | | | | | | | | | |
| 4 | randomised trials | | not serious | not serious | not serious | serious^c^ | none | 66 | 64 | SMD **0.46 higher** (0.11 higher to 0.81 higher) | ⨁⨁⨁◯ Moderate^c^ | IMPORTANT |
| **STS-5(s)** | | | | | | | | | | | | |
| 2 | randomised trials | | not serious | not serious | not serious | serious^d^ | none | 25 | 22 | MD **1.19 lower** (4.93 lower to 2.54 higher) | ⨁⨁⨁◯ Moderate^d^ | IMPORTANT |
| **STS-10(s)** | | | | | | | | | | | | |
| 3 | randomised trials | | serious^a^ | not serious | not serious | serious^d^ | none | 45 | 42 | MD **3.59 lower** (8.65 lower to 1.46 higher) | ⨁⨁◯◯ Low^a,d^ | IMPORTANT |
| **STS-60(s)** | | | | | | | | | | | | |
| 4 | randomised trials | | serious^a^ | serious^e^ | not serious | serious^d^ | none | 54 | 54 | MD **3.04 higher** (0.82 lower to 6.9 higher) | ⨁◯◯◯ Very low^a,d,e^ | IMPORTANT |
| **6MWT(meters)** | | | | | | | | | | | | |
| 4 | randomised trials | | serious^a^ | not serious | not serious | not serious | none | 85 | 90 | MD **74.77 higher** (40.55 higher to 108.99 higher) | ⨁⨁⨁◯ Moderate^a^ | IMPORTANT |
| **Heart rate - HR(min)** | | | | | | | | | | | | |
| 3 | randomised trials | | serious^a^ | not serious | not serious | serious^c^ | none | 97 | 96 | MD **3.64 lower** (6.96 lower to 0.31 lower) | ⨁⨁◯◯ Low^a,c^ | IMPORTANT |
| **Respiratory rate - RR(min)** | | | | | | | | | | | | |
| 3 | randomised trials | | serious^a^ | serious^b^ | not serious | serious^d^ | none | 97 | 96 | MD **0.12 higher** (1.24 lower to 1.48 higher) | ⨁◯◯◯ Very low^a,b,d^ | IMPORTANT |
| **Systolic blood pressure - SBP(mmHg)** | | | | | | | | | | | | |
| 3 | randomised trials | | serious^a^ | serious^e^ | not serious | serious^d^ | none | 97 | 96 | MD **0.54 lower** (11.05 lower to 9.97 higher) | ⨁◯◯◯ Very low^a,d,e^ | IMPORTANT |
| **Diastolic blood pressure - DBP(mmHg)** | | | | | | | | | | | | |
| 3 | randomised trials | | serious^a^ | not serious | not serious | serious^d^ | none | 97 | 96 | MD **0.66 lower** (3.41 lower to 2.1 higher) | ⨁⨁◯◯ Low^a,d^ | IMPORTANT |
| **SpO₂(%)** | | | | | | | | | | | | |
| 3 | randomised trials | | serious^a^ | not serious | not serious | serious^e^ | none | 97 | 96 | MD **0.4 higher** (0.04 higher to 0.75 higher) | ⨁⨁◯◯ Low^a,e^ | IMPORTANT |
| **Physical Functioning-PF** | | | | | | | | | | | | |
| 3 | randomised trials | | serious^a^ | not serious | not serious | serious^c^ | none | 61 | 61 | MD **11.68 higher** (5.38 higher to 17.98 higher) | ⨁⨁◯◯ Low^a,c^ | IMPORTANT |
| **Role-Physical-RP** | | | | | | | | | | | | |
| 2 | randomised trials | | serious^a^ | not serious | not serious | serious^c^ | none | 41 | 41 | MD **18.41 higher** (9.92 higher to 26.91 higher) | ⨁⨁◯◯ Low^a,c^ | IMPORTANT |
| **Bodily Pain-BP** | | | | | | | | | | | | |
| 2 | randomised trials | | serious^a^ | not serious | not serious | serious^c^ | none | 41 | 41 | MD **8.75 higher** (2.12 higher to 15.37 higher) | ⨁⨁◯◯ Low^a,c^ | IMPORTANT |
| **General Health-GH** | | | | | | | | | | | | |
| 2 | randomised trials | | serious^a^ | not serious | not serious | serious^c^ | none | 41 | 41 | MD **9.23 higher** (3.77 higher to 14.68 higher) | ⨁⨁◯◯ Low^a,c^ | IMPORTANT |
| **Vitality-VT** | | | | | | | | | | | | |
| 3 | randomised trials | | serious^a^ | not serious | not serious | serious^e^ | none | 61 | 61 | MD **13.17 higher** (6.98 higher to 19.36 higher) | ⨁⨁◯◯ Low^a,e^ | IMPORTANT |
| **Social Functioning-SF** | | | | | | | | | | | | |
| 2 | randomised trials | | serious^a^ | not serious | not serious | serious^d^ | none | 41 | 41 | MD **6.23 higher** (0.86 lower to 13.33 higher) | ⨁⨁◯◯ Low^a,d^ | IMPORTANT |
| **Role-Emotional-RE** | | | | | | | | | | | | |
| 2 | randomised trials | | serious^a^ | not serious | not serious | serious^d^ | none | 41 | 41 | MD **6.89 higher** (0.18 lower to 13.97 higher) | ⨁⨁◯◯ Low^a,d^ | IMPORTANT |
| **Mental Health - MH** | | | | | | | | | | | | |
| 2 | randomised trials | | serious^a^ | not serious | not serious | serious^c^ | none | 41 | 41 | MD **19.14 higher** (8.8 higher to 29.47 higher) | ⨁⨁◯◯ Low^a,c^ | IMPORTANT |

**CI:** confidence interval; **MD:** mean difference; **SMD:** standardised mean difference

a. The proportion of medium- and high-risk studies is greater than 33.3%

b. High heterogeneity was observed (I² ranging from 50% to 90%), and its source could not be identified through subgroup or sensitivity analyses.

c. The total sample size included in the study was limited, the 95% CI for the pooled effects were wide, and the precision of the results was insufficient

d. The combined effect was not statistically significant (P > 0.05); the 95% CI straddled the null hypothesis line, the results are imprecise.

e. Mild heterogeneity was largely attributable to one study; its removal eliminated heterogeneity but substantially altered the overall result.
