# Supplementary material for: Effectiveness and Safety of Virtual Reality Interventions for Symptom Management in Adults Undergoing Hemodialysis: Systematic Review and Meta-Analysis of Randomized Controlled Trials
Source: J Med Internet Res. 2026 Sep 28;28:e102331. doi: 10.2196/102331 (PMC13618410; doi:10.2196/102331)
Supplement: Supplementary file 4 [file jmir-v28-e102331-s004.docx]

**Table S1.** Sensitivity Analysis Across Two Models

| **Influencing Factors** | **random-effect model** | | | **fixed-effect model** | | | **Result consistency** |
| --- | --- | --- | --- | --- | --- | --- | --- |
|  | ***P*** | **SMD/MD** | **95%CI** | ***P*** | **SMD/MD** | **95%CI** |  |
| Pain | < 0.001 | -2.44 | -2.91 ~ -1.97 | < 0.001 | -2.38 | -2.63 ~ -2.14 | Consistent |
| State anxiety | 0.20 | -9.64 | -24.43 ~ 5.15 | < 0.001 | -19.20 | -20.57 ~ -17.82 | Inconsistent |
| Trait anxiety | 0.01 | -7.72 | -13.72 ~ -1.71 | < 0.001 | -9.58 | -11.03 ~ -8.14 | Consistent |
| Depressive symptoms | 0.08 | -3.62 | -7.70 ~ 0.46 | 0.04 | -3.48 | -6.77 ~ -0.18 | Inconsistent |
| TUG | 0.09 | -1.28 | -2.73 ~ 0.18 | 0.09 | -1.28 | -2.73 ~ 0.18 | Consistent |
| Gait speed | 0.01 | 0.46 | 0.11 ~ 0.81 | 0.01 | 0.46 | 0.11 ~ 0.81 | Consistent |
| STS-5 | 0.62 | -1.05 | -5.24 ~ 3.13 | 0.53 | -1.19 | -4.93 ~ 2.54 | Consistent |
| STS-10 | 0.16 | -3.59 | -8.65~ 1.46 | 0.16 | -3.59 | -8.65~ 1.46 | Consistent |
| STS-60 | 0.29 | 2.57 | -2.20 ~ 7.34 | 0.12 | 3.04 | -0.82~ 6.90 | Consistent |
| 6MWT | 0.002 | 76.95 | 27.71 ~ 126.18 | < 0.001 | 74.77 | 40.55 ~ 108.99 | Consistent |
| Heart rate | 0.03 | -3.64 | -6.96 ~ -0.31 | 0.03 | -3.64 | -6.96 ~ -0.31 | Consistent |
| Respiratory rate | 0.87 | 0.12 | -1.24 ~ 1.48 | 0.53 | -0.21 | -0.88 ~ 0.45 | Consistent |
| Systolic blood pressure | 0.92 | -0.54 | -11.05 ~ 9.97 | 0.28 | -2.94 | -8.25 ~ 2.37 | Consistent |
| Diastolic blood pressure | 0.64 | -0.66 | -3.41~2.10 | 0.64 | -0.66 | -3.41~2.10 | Consistent |
| Peripheral oxygen saturation | 0.06 | 0.42 | -0.02~0.85 | 0.03 | 0.40 | 0.04~0.75 | Inconsistent |
| Physical functioning | < 0.001 | 11.68 | 5.38 ~ 17.98 | < 0.001 | 11.68 | 5.38 ~ 17.98 | Consistent |
| Role-physical | < 0.001 | 18.41 | 9.92 ~ 26.91 | < 0.001 | 18.41 | 9.92 ~ 26.91 | Consistent |
| Bodily pain | 0.01 | 8.75 | 2.12 ~ 15.37 | 0.01 | 8.75 | 2.12 ~ 15.37 | Consistent |
| General health | < 0.001 | 9.23 | 3.77 ~ 14.68 | < 0.001 | 9.23 | 3.77 ~ 14.68 | Consistent |
| Vitality | < 0.001 | 13.17 | 6.98 ~ 19.36 | < 0.001 | 13.17 | 6.98 ~ 19.36 | Consistent |
| Social functioning | 0.09 | 6.23 | -0.86 ~ 13.33 | 0.09 | 6.23 | -0.86 ~ 13.33 | Consistent |
| Role-emotional | 0.06 | 6.89 | -0.18 ~ 13.97 | 0.06 | 6.89 | -0.18 ~ 13.97 | Consistent |
| Mental health | < 0.001 | 19.14 | 8.80 ~ 29.47 | < 0.001 | 19.14 | 8.80 ~ 29.47 | Consistent |

Note. Gait speed was reported as standardized mean difference (SMD), and other outcomes were presented as mean difference (MD).
