# Supplementary material for: Effectiveness and Safety of Virtual Reality Interventions for Symptom Management in Adults Undergoing Hemodialysis: Systematic Review and Meta-Analysis of Randomized Controlled Trials
Source: J Med Internet Res. 2026 Sep 28;28:e102331. doi: 10.2196/102331 (PMC13618410; doi:10.2196/102331)
Supplement: Supplementary file 5 [file jmir-v28-e102331-s005.docx]

**Figure S1.** Sensitivity analysis of the effect of virtual reality interventions on arteriovenous fistula cannulation pain.

Note. This sensitivity analysis (excluding the study by Namazinia et al) is presented in Section 3.5.1 on page 23 of the main manuscript.

**Figure S2.** Sensitivity analysis of the effect of virtual reality interventions on state anxiety.

Note. This sensitivity analysis (excluding the study by Güler et al) is presented in Section 3.5.2 on page 25 of the main manuscript.

**Figure S3.** Sensitivity analysis of the effect of VR-based exercise on gait speed.

Note. (A)Sensitivity analysis after excluding the study by Segura‑Ortí ① et al.

(B) Sensitivity analysis after excluding the study by Segura‑Ortí ② et al.

(C) Sensitivity analysis after excluding the study by Martínez‑Olmos et al.

(D) Sensitivity analysis after excluding the study by Maynard et al.

These sensitivity analyses are presented in Section 3.5.3 on page 27 of the main manuscript.

**Figure S4.** Sensitivity analysis of the effect of VR-based exercise on 10-second sit-to-stand test.

Note. (A)Sensitivity analysis after excluding the study by Segura‑Ortí ① et al.

(B) Sensitivity analysis after excluding the study by Segura‑Ortí ② et al.

(C) Sensitivity analysis after excluding the study by Martínez‑Olmos et al.

These sensitivity analyses are presented in Section 3.5.3 on page 28 of the main manuscript.

**Figure S5.** Sensitivity analysis of the effect of VR-based exercise on 60-second sit-to-stand test.

Note. This sensitivity analysis (excluding the study by Schinner et al) is presented in Section 3.5.3 on page 28 of the main manuscript.

**Figure S6.** Sensitivity analysis of the effect of VR-based exercise on 6-minute walk test.

Note. (A) Sensitivity analysis after excluding the study by Turon-Skrzypinska et al.

(B)Sensitivity analysis after excluding the study by Segura‑Ortí ① et al.

(C) Sensitivity analysis after excluding the study by Segura‑Ortí ② et al.

(D) Sensitivity analysis after excluding the study by Martínez‑Olmos et al.

These sensitivity analyses are presented in Section 3.5.3 on page 29 of the main manuscript.

**Figure S7.** Sensitivity analysis of the effect of VR-based exercise on heart rate.

Note. This sensitivity analysis (excluding the study by Mohamed et al) is presented in Section 3.5.4 on page 31 of the main manuscript.

**Figure S8.** Sensitivity analysis of the effect of VR-based exercise on respiratory rate.

Note. (A)Sensitivity analysis after excluding the study by Şen et al.

(B) Sensitivity analysis after excluding the study by Maynard et al.

(C) Sensitivity analysis after excluding the study by Mohamed et al.

These sensitivity analyses are presented in Section 3.5.3 on page 31 of the main manuscript.

**Figure S9.** Sensitivity analysis of the effect of VR-based exercise on systolic blood pressure.

Note. This sensitivity analysis (excluding the study by Şen et al) is presented in Section 3.5.4 on page 32 of the main manuscript.

**Figure S10.** Sensitivity analysis of the effect of VR-based exercise on diastolic blood pressure.

Note. (A)Sensitivity analysis after excluding the study by Şen et al.

(B) Sensitivity analysis after excluding the study by Maynard et al.

(C) Sensitivity analysis after excluding the study by Mohamed et al.

These sensitivity analyses are presented in Section 3.5.3 on page 32-33 of the main manuscript.

**Figure S11.** Sensitivity analysis of the effect of VR-based exercise on peripheral oxygen saturation.

Note. This sensitivity analysis (excluding the study by Mohamed et al) is presented in Section 3.5.4 on page 33 of the main manuscript.

**Figure S12.** Sensitivity analysis of the effect of VR-based exercise on physical functioning.

Note. This sensitivity analysis (excluding the study by Feng et al) is presented in Section 3.5.5 on page 35 of the main manuscript.

**Figure S13.** Sensitivity analysis of the effect of VR-based exercise on vitality.

Note. This sensitivity analysis (excluding the study by Feng et al) is presented in Section 3.5.5 on page 36-37 of the main manuscript.
