# Supplementary material for: Effectiveness and Safety of Virtual Reality Interventions for Symptom Management in Adults Undergoing Hemodialysis: Systematic Review and Meta-Analysis of Randomized Controlled Trials
Source: J Med Internet Res. 2026 Sep 28;28:e102331. doi: 10.2196/102331 (PMC13618410; doi:10.2196/102331)
Supplement: Supplementary file 6 [file jmir-v28-e102331-s006.docx]

**Table S1.** Sensitivity analyses excluding studies by the Segura-Ortí team for physical function outcomes.

| Outcome | Primary analysis | Excluding Segura-Ortí 2019① | Excluding Segura-Ortí 2019② | Excluding all Segura-Ortí studies | Interpretation |
| --- | --- | --- | --- | --- | --- |
| Gait speed | 0.46 [0.11, 0.81], P=.01 | 0.51 [0.09, 0.93], P=.02 | 0.50 [0.12, 0.87], P=.009 | 0.58 [0.11, 1.05], P=.02 | Remained significant |
| STS-5 | -1.19 [-4.93, 2.54], P=.53; Figure | Not pooled; only one study remained | | | Insufficient studies |
| STS-10 | -3.59 [-8.65, 1.46], P=.16 | -3.48 [-9.76, 2.80], P=.28 | -4.96 [-10.49, 0.58], P=.08 | Not pooled; only one study remained | Remained nonsignificant |
| STS-60 | 3.04 [-0.82, 6.90], P=.12 | 1.67 [-3.07, 6.42], P=.49 | 3.54[-0.61, 7.68], P=.09 | 0.73 [-9.58, 11.04], P=.89 | Remained nonsignificant |
| 6MWT | 74.77 [40.55, 108.99], P＜.0001 | 66.15 [29.03, 103.27], P=.0005 | 88.48 [51.25, 125.72], P＜.00001 | 80.90 [39.83, 121.97], P=.0001 | Remained significant |

Note. Gait speed was synthesized using standardized mean difference (SMD); all other physical function outcomes were pooled as mean difference (MD). All pooled estimates are presented with corresponding 95% confidence intervals and P values. “Not pooled” indicates fewer than two eligible trials remained after exclusion, precluding quantitative meta-analysis. VR = virtual reality; STS-5 = five-times sit-to-stand test; STS-10 = ten-times sit-to-stand test; STS-60 = 60-second sit-to-stand test; 6MWT = six-minute walk test.

**Figure S1.** Forest plots for sensitivity analyses of gait speed after excluding Segura-Ortí et al. (2019) trials.

Note. (A) Primary analysis; (B) excluding Segura-Ortí 2019①; (C) excluding Segura-Ortí 2019②; (D) excluding all Segura-Ortí studies.

**Figure S2.** Forest plots for sensitivity analyses of STS-5 after excluding Segura-Ortí et al. (2019) trials.

Note. Forest plot shows the pooled effect of virtual reality intervention versus usual care on STS-5 in the primary analysis. Sensitivity analyses excluding Segura-Ortí studies were not pooled because only one eligible study remained.

**Figure S3.** Forest plots for sensitivity analyses of STS-10 after excluding Segura-Ortí et al. (2019) trials.

Note. (A) Primary analysis; (B) excluding Segura-Ortí 2019①; (C) excluding Segura-Ortí 2019②. The analysis excluding all Segura-Ortí studies was not pooled because only one eligible study remained.

**Figure S4.** Forest plots for sensitivity analyses of STS-60 after excluding Segura-Ortí et al. (2019) trials.

Note. (A) Primary analysis; (B) excluding Segura-Ortí 2019①; (C) excluding Segura-Ortí 2019②; (D) excluding all Segura-Ortí studies.

**Figure S5.** Forest plots for sensitivity analyses of 6MWT after excluding Segura-Ortí et al. (2019) trials.

Note. (A) Primary analysis; (B) excluding Segura-Ortí 2019①; (C) excluding Segura-Ortí 2019②; (D) excluding all Segura-Ortí studies.
